# Improved demodulated phase signal resolution for carrier signals with small modulation index by clipping and synchronous sampling for heterodyne interferometers

**DOI:** 10.1038/s41598-023-35000-2

**Published:** 2023-05-26

**Authors:** M. Yu, M. Schewe, G. Bauer, C. Rembe

**Affiliations:** Department of Applied Metrology, Institute of Electrical Information Technology, TU Clausthal, Clausthal-Zellerfeld, Germany

**Keywords:** Optical sensors, Imaging and sensing

## Abstract

Digitization of phase-modulated carrier signals with a commercially available analog-to-digital converter (ADC) is a common task in many communication and sensor applications. ADCs deliver phase-modulated digital carrier signals, which are numerically demodulated in order to extract the relevant information. However, the limited dynamic ranges of available ADCs limit the carrier-to-noise ratio of carrier signals after digitization. Correspondingly, the resolution of the demodulated digital signal is degraded. We demonstrate a sampling method with a simple demodulation scheme for phase-modulated signals with a small modulation index. Our new scheme overcomes the limitation due to digital noise defined by the ADC. Through simulations and experiments, we provide evidence that our method can improve the resolution of the demodulated digital signal significantly, when the carrier-to-noise ratio of phase-modulated signals is limited by digital noise. We employ our sampling and demodulation scheme to solve the problem of a possible degradation of measurement resolution after digital demodulation in heterodyne interferometers measuring small vibration amplitudes.

## Introduction

Phase modulation^1^ (PM) is applied in many fields, such as satellite communication systems^2^, microwave Doppler radar systems^3^, interferometry^4^, laser Doppler vibrometers (LDV)^5^, and phase modulated spectrometers^6^. The sensitivity of communication and measurement systems depends on the carrier-to-noise ratio (CNR) of the carrier signal for PM^7^.

Nowadays, optical frequency division and regenerative frequency dividers techniques implement ultra-low phase noise oscillators. They can provide a stable and clean carrier signal with a high CNR for PM. The reported phase noise relative to the carrier is lower than $- \,180\;{\text{dBc}}/{\text{Hz}}$ for an 8 GHz frequency microwave at a modulation frequency of 1 MHz^8^ and lower than $- \,170 \;{\text{dBc}}/{\text{Hz}}$ for radio frequencies in the megahertz range with a modulation bandwidth of 100 kHz^9^. When using an optical beam as a carrier for PM, the phase noise of the narrow-linewidth laser beam at a photodetector is limited by photon shot noise^10^ and even beyond the photon shot-noise-limit by application of squeezed states of light^11,12^. Optical carriers with high CNR due to superior phase sensitivity are suitable for some applications of PM signals with small modulation indices, such as the measurements of micro-vibration on micro-electro-mechanical systems^7,13^, basilar membrane vibration^14^ and surface acoustic wave^15^.

The demodulation without information loss for the optical or electrical signal with a high CNR and small modulation indices requires a sensitive demodulation scheme. Some established analog demodulation schemes, e.g. the delay-line frequency discriminator or the phase detector method, employ reference source and phase-locked loop (PLL) circuit techniques^16^, but suffer from the aging of electronic devices, thermal drift, nonlinearity etc.^17^ and do not achieve the noise limits of digital demodulation. The digital in-phase and quadrature (IQ) demodulation techniques based on digital signal processor utilize the direct digitization of the carrier signal and numerical demodulation methods instead of complex electronic arrangements (mixer, PLL circuit, voltage controlled oscillator etc.) and achieve a high accuracy, low cost and flexible signal processing.

Usually, the users should choose analog-to-digital converter (ADC) hardware chips or integrated modules with the appropriate CNR to satisfy the demand. For example, the optical signal at the photodetector of an eye-save LDV (the measuring light with the wavelength of $\lambda = 1550 \;{\text{nm}}$ and the power of $P_{{\text{m}}} = 10\; {\text{mW}}$) was experimentally demonstrated at $CNR_{{{\text{dB}}}} = 167.5{ }\;{\text{dB}}$ in a resolution bandwidth (RBW) of 1 Hz^13^ close to the theoretical limit. To obtain a digital signal with $CNR_{{{\text{dB}}}} = 167.5{\text{ dB}}$, the number of bits of the ADC with a sampling rate $f_{{\text{s}}} = 250 \;{\text{MSps}}$ is chosen to be 16 bits or higher. The delta-sigma ADC has the highest resolution up to 24 bits and a relatively low sample rate from kilo samples per second (kSps) to mega samples per second (MSps)^18^. This is obviously not applicable to LDV signals with a typical carrier at 40 MHz and carriers for ultra-high-frequency applications that can reach GHz frequencies^19,20^. Oversampling is another solution to reduce quantization noise. The flash ADCs have the highest sampling rates up to giga samples per second (GSps) due to their parallel structure but with a resolution limited to no more than 8 bits due to nonlinearity^21^. Although some SAR (successive-approximation-register) ADCs have the potential to achieve 16-bit and 250 MSps or better CNR, it means higher costs and larger power consumption^22^. Therefore, many researchers worked to achieve high resolution on an existing ADC with lower resolution. However, most digital calibration techniques and adding dithering to ADC channels^23^ only improve the spurious-free dynamic range of the signal, but the noise power spectral density of the quantized noise remains unchanged. The averaging technique^24,25^ converts the same input voltage several times to reduce the quantization noise, but at the cost of a significant reduction in conversion speed, which results in it being more suitable for slowly varying signals. The cross-correlation of the ADC's two input channels^26^ allow improvement of the noise floor but it is computationally expensive to calculate. Decreasing the input range of the ADC can directly improve the resolution of the ADC, but this will suffer from the interference of clipping on the signal^27^. Clipping is a non-linear process that usually brings an attenuation of the energy within the demodulation bandwidth and numerous high frequency harmonics^28,29^. Based on the limited dynamic range of state-of-the-art ADCs reported in^30^, PM signals with a high CNR (e.g. higher than 167.5 dB) at high frequency or ultra-high frequency encounter the challenge of resolution degradation during direct sampling. In this paper, we present a sampling and processing method for a PM signal with small modulation index.

The scientific hypothesis of our paper is that the achievable noise equivalent phase deviation of a carrier signal is not limited by the possible CNR for the ADC, if the sine carrier signal is digitized in the full range without clipping.

The theoretical CNR in dB of a sine function digitized with an effective *X*-bit ADC with a sampling rate $f_{{\text{s}}}$ for RBW 1 Hz can be denoted as $CNR_{{{\text{qn}}, {\text{dB}}}} = 10 \cdot {\text{log}}_{10} \left( {CNR_{{{\text{qn}}}} } \right) = 6.02 \cdot X + 1.76 + 10\log \frac{{f_{{\text{s}}} }}{2}$^31^. The phase resolution $\Delta \varphi$ of the demodulated phase $\varphi \left( t \right)$ of such a digitized sine carrier signal in $1\;{\text{Hz}}$ bandwidth is given by  $\Delta \varphi = \sqrt{ {2 / CNR_{{{\text{qn}}}} } }$. Thus, an 8-bit ADC with a sampling rate $f_{{\text{s}}} = 250\; {\text{mega samples per second}} \left( {{\text{MSps}}} \right)$ can only achieve the CNR limited by quantization noise of $131{\text{ dB}}$ in a RBW of 1 Hz and consequently the phase resolution is $\Delta \varphi \approx 0.4 \;\upmu {\text{rad}}$. In a heterodyne interferometer with a wavelength of $\lambda = 1550 \;{\text{nm}}$ that would correspond to a displacement resolution in 1 Hz bandwidth of $\Delta s = \frac{\lambda }{4\pi }\Delta \varphi = 50\; {\text{fm}}$. This limitation is commonly accepted as the resolution limit of heterodyne interferometers. In this paper, we show a method that can breach this limit for a given ADU if the sampling can be synchronized to the zero crossings of the unmodulated carrier signal. Clipping and synchronous sampling with a simple demodulation scheme allows to resolve small sidebands with the full bit resolution of the ADC.

In the paper, we demonstrate that the SNR of the demodulated phase for an ADC with insufficient bit-resolution can be increased with clipping compared to conventional sampling technique without clipping. Since our motivation for the work presented in this paper is originated by the requirements of heterodyne interferometry, we present the application of our method to a heterodyne interferometer (HI) in the experimental section. Our method presented in this paper will help to break ADC noise limitation on the digital demodulation and to make photon shot-noise limited sensitivity possible for heterodyne interferometry. However, the method is applicable to all broadband, PM-modulated carrier signals with small maximum phase deviations and could potentially be applied in other technical fields.

## Methods

### Digitization of a clipped PM signal with synchronous sampling

**Figure 1:**
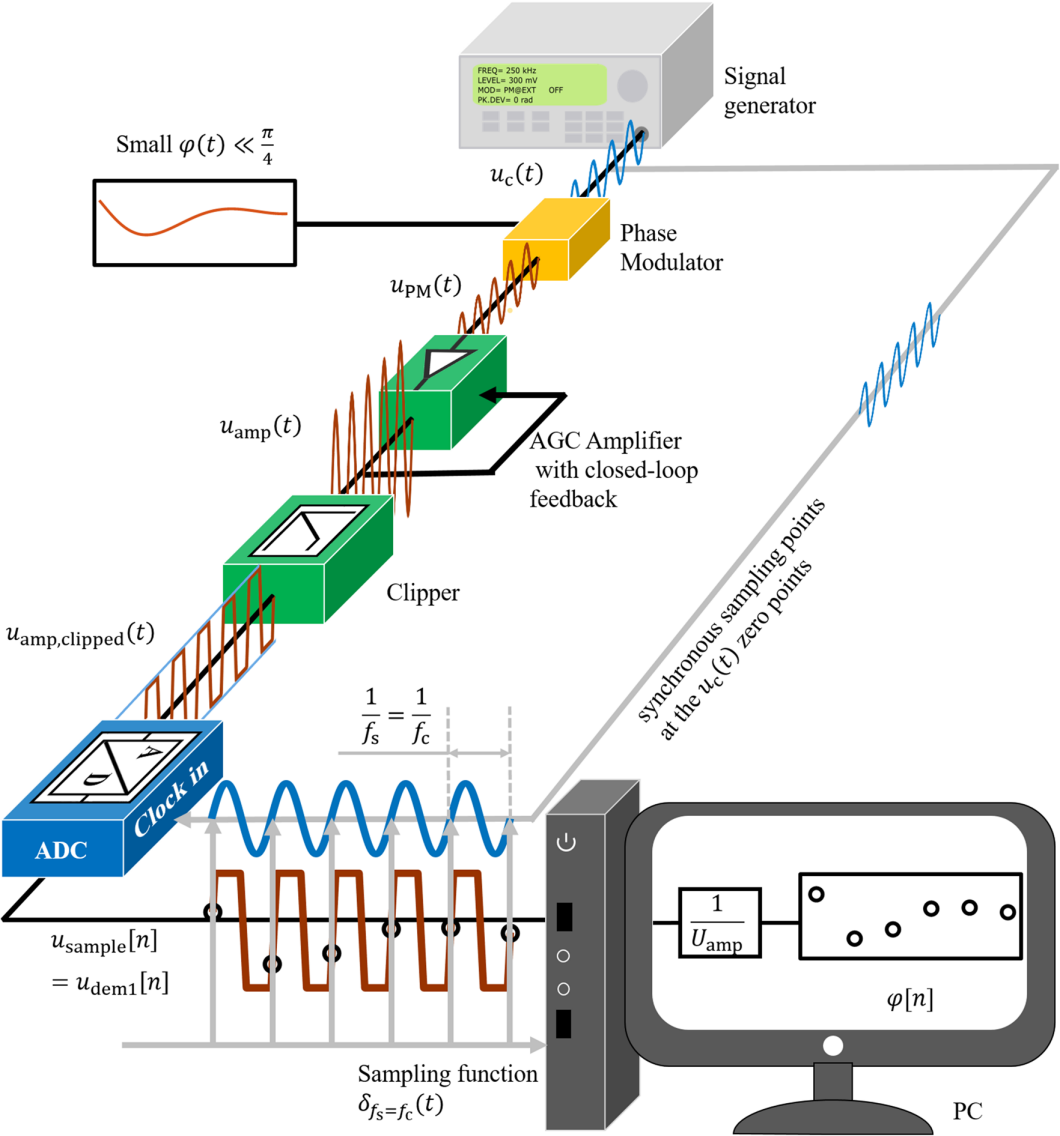
Schematic of digitization of a clipped PM signal with synchronous sampling. The carrier $u_{{\text{c}}} \left( t \right)$ generated by the signal generator is phase modulated by the small information signal $\varphi \left( t \right)$ into $u_{{{\text{PM}}}} \left( t \right)$. The operation of amplification to $u_{{{\text{amp}}}} \left( t \right)$ followed by clipping to $u_{{{\text{amp}},{\text{ clipped}}}} \left( t \right)$ is to fit the input range $\pm K$ of the analog-to-digital converter (ADC). The synchronous sampling points are located at the unmodulated carrier $u_{{\text{c}}} \left( t \right)$ zero points by the sampling functions $\delta_{{f_{{\text{s}}} = f_{{\text{c}}} }} \left( t \right)$ with the sampling frequency $f_{{\text{s}}}$ and the carrier frequency $f_{{\text{c}}}$. The amplifier with automatic gain control (AGC) provides a stable amplified carrier voltage amplitude $U_{{{\text{amp}}}}$ while amplifying the signal power. Consequently, the sampling points achieve a linear relationship between the discrete small modulation signal $\varphi \left[ n \right]$ and the demodulated signal $u_{{{\text{dem}}1}} \left[ n \right] = u_{{{\text{sample}}}} \left[ n \right] = \varphi \left[ n \right]U_{{{\text{amp}}}}$ and, thus, the demodulated phase $\varphi \left[ n \right]$ can be simply obtained from the $u_{{{\text{dem}}1}} \left[ n \right]$ by the factor $\frac{1}{{U_{{{\text{amp}}}} }}$ in the computer.

We consider the following scenario. A signal generator, as shown in Fig. 1, generates a carrier voltage signal

1 $$u_{c} \left( t \right) = U_{c} \sin \left( {2\uppi f_{c} t} \right).$$

Here, $U_{{\text{c}}}$ is the amplitude of the voltage signal ${ }u_{{\text{c}}} \left( t \right)$ and $f_{{\text{c}}}$ is the carrier frequency. The carrier signal $u_{{\text{c}}} \left( t \right)$ is phase modulated into

2 $$u_{{{\text{PM}}}} \left( t \right) = U_{{\text{c}}} \sin \left( {2{\uppi }f_{{\text{c}}} t + \varphi \left( t \right)} \right),$$

where $\varphi \left( t \right) = M\varphi_{{{\text{norm}}}} \left( t \right){ }$ is the modulation phase with the phase modulation index $M = \varphi_{{{\text{max}}}}$ as maximum phase stroke and the normalized, broadband modulation signal $\varphi_{{{\text{norm}}}} \left( t \right)$.

Because of the sum identities for sine, Eq. (2) can further be expanded to

3 $$u_{{{\text{PM}}}} \left( t \right) = U_{{\text{c}}} \sin (2{\uppi }f_{{\text{c}}} t)\cos \varphi \left( t \right) + U_{{\text{c}}} \cos (2{\uppi }f_{{\text{c}}} t)\sin \varphi \left( t \right).$$

When the modulation index $M$ is very small, namely ${ }M \ll 1$ and $\varphi \left( t \right) \ll 1$, Eq. (3) can be approximated by

4 $$u_{{{\text{PM}}}} \left( t \right) \approx U_{{\text{c}}} \sin (2{\uppi }f_{{\text{c}}} t) + \varphi \left( t \right)U_{{\text{c}}} \cos (2{\uppi }f_{{\text{c}}} t)$$

with the small-angle approximations $\cos \varphi \left( t \right) \approx 1 \;{\text{and}}\;\sin \varphi \left( t \right) \approx \varphi \left( t \right)$.

An electronic amplifier with automatic gain control (AGC) converts a weak electrical signal into an output signal strong enough to be noise-tolerant and strong enough for further processing. The AGC ensures that the amplified carrier signal $u_{{{\text{amp}}}} \left( t \right)$ has a well-defined amplitude $U_{{{\text{amp}}}}$. Figure 1 shows the signal transmission chain. The phase modulated signal $u_{{{\text{PM}}}} \left( t \right)$ is amplified to the signal

5 $$u_{{{\text{amp}}}} \left( t \right) = U_{{{\text{amp}}}} \sin (2{\uppi }f_{{\text{c}}} t) + \varphi \left( t \right)U_{{{\text{amp}}}} \cos (2{\uppi }f_{{\text{c}}} t)$$

with a defined amplitude $U_{{{\text{amp}}}}$ by an AGC. The AGC ensured that the amplitude $U_{{{\text{amp}}}}$ of the amplified signal $u_{{{\text{amp}}}} \left( t \right)$ is larger than the input range thresholds $+ K$ and $- K$ of the ADC ($U_{{{\text{amp}}}} > K$). In this case, a clipper is placed in front of the ADC to limit the maximum amplitude of the amplified signal $u_{{{\text{amp}}}} \left( t \right)$ to $K$ and to avoid overload of the ADC. The signal will be clipped, as shown in Fig. 1. The clipped PM signal is expressed by

6 $$u_{{{\text{amp}},{\text{ clipped}}}} \left( t \right) = \left\{ {\begin{array}{*{20}c} K & {u_{{{\text{amp}}}} \left( t \right) > K} \\ {u_{{{\text{amp}}}} \left( t \right)} & { - K \le u_{{{\text{amp}}}} \left( t \right) \le K} \\ { - K} & {u_{{{\text{amp}}}} \left( t \right) < - K} \\ \end{array} } \right.,$$

where the unclipped part still contains information.

In order to efficiently use the unclipped part of the clipped PM signal $u_{{{\text{amp}},{\text{ clipped}}}} \left( t \right)$, we propose a synchronous sampling method whose sampling points are always located at the zero crossings of a rising edge of the unmodulated carrier $u_{{\text{c}}} \left( t \right)$ as shown in Fig. 1. The $,N \in {\mathbb{N}}^{ + }$ sampling time points $t\left[ n \right],\left( {n \in 1,2, \ldots N} \right)$ of the ADC are synchronized to the zero crossings $u_{{\text{c}}} \left( {t\left[ n \right]} \right) = U_{{\text{c}}} \sin (2{\uppi }f_{{\text{c}}} t\left[ n \right]) = 0$ at a rising edge of the unmodulated carrier $u_{{\text{c}}} \left( t \right)$. Thus, the amplified modulated carrier signal $u_{{{\text{amp}}}} \left( t \right)$ can be approximated at the sampling time points $t\left[ n \right]$ as $u_{{{\text{amp}}}} \left( {t\left[ n \right]} \right) = U_{{{\text{amp}}}} \sin (2{\uppi }f_{{\text{c}}} t\left[ n \right]) + \varphi \left( {t\left[ n \right]} \right)U_{{{\text{amp}}}} \cos( 2{\uppi }f_{{\text{c}}} t\left[ n \right])\approx \varphi \left( {t\left[ n \right]}\right)U_{{{\text{amp}}}}$. Here, it is assumed that the maximal possible phase stroke is small enough to allow a linear approximation of $u_{{{\text{amp}}}} \left( t \right)$ at the zero crossings in respect to Eq. (5). At the sample time points $\left[ n \right]$, the clipped signal $u_{{{\text{amp}},{\text{ clipped}}}} \left( {t\left[ n \right]} \right)$ and the amplified modulated carrier signal $u_{{{\text{amp}}}} \left( {t\left[ n \right]} \right)$ are equal $u_{{{\text{amp}},{\text{ clipped}}}} \left( {t\left[ n \right]} \right) = u_{{{\text{amp}}}} \left( {t\left[ n \right]} \right) \le K$. Therefore, according to Eq. (5),

7 $$u_{{{\text{amp}},{\text{clipped}}}} \left( {t\left[ n \right]} \right) \approx \varphi \left( {t\left[ n \right]} \right)U_{{{\text{amp}}}}$$

is valid. The sampled signal $u_{{{\text{sample}}}} \left( {t\left[ n \right]} \right)$ of the clipped phase-modulated carrier signal $u_{{{\text{amp}},{\text{clipped}}}} \left( {t\left[ n \right]} \right)$ at time points $t\left[ n \right]$ yields the discrete sampled signal $u_{{{\text{sample}}}} \left[ n \right]$ and the discrete modulated phase signal $\varphi \left[ n \right] = \frac{{u_{{{\text{sample}}}} \left[ n \right]}}{{U_{{{\text{amp}}}} }}$ for sampling frequency $f_{{\text{s}}} = f_{{\text{c}}}$. For sampling frequency $f_{{\text{s}}} = f_{{\text{c}}}$ the signal $u_{{{\text{sample}}}} \left[ n \right]$ corresponds automatically to the demodulated signal $u_{{{\text{dem}}1}} \left[ n \right] = u_{{{\text{sample}}}} \left[ n \right]$. The linear relationship between the discrete sampled signal $u_{{{\text{dem}}1}} \left[ n \right]$ and the discrete modulated phase signal $\varphi \left[ n \right] = \frac{{u_{{{\text{dem}}1}} \left[ n \right]}}{{U_{{{\text{amp}}}} }}$ makes it possible to directly demodulate a sufficiently clipped PM signal with low modulation index $M \ll 1$ by a constant coefficient $U_{{{\text{amp}}}}$ as presented in Fig. 1.

**Figure 2:**
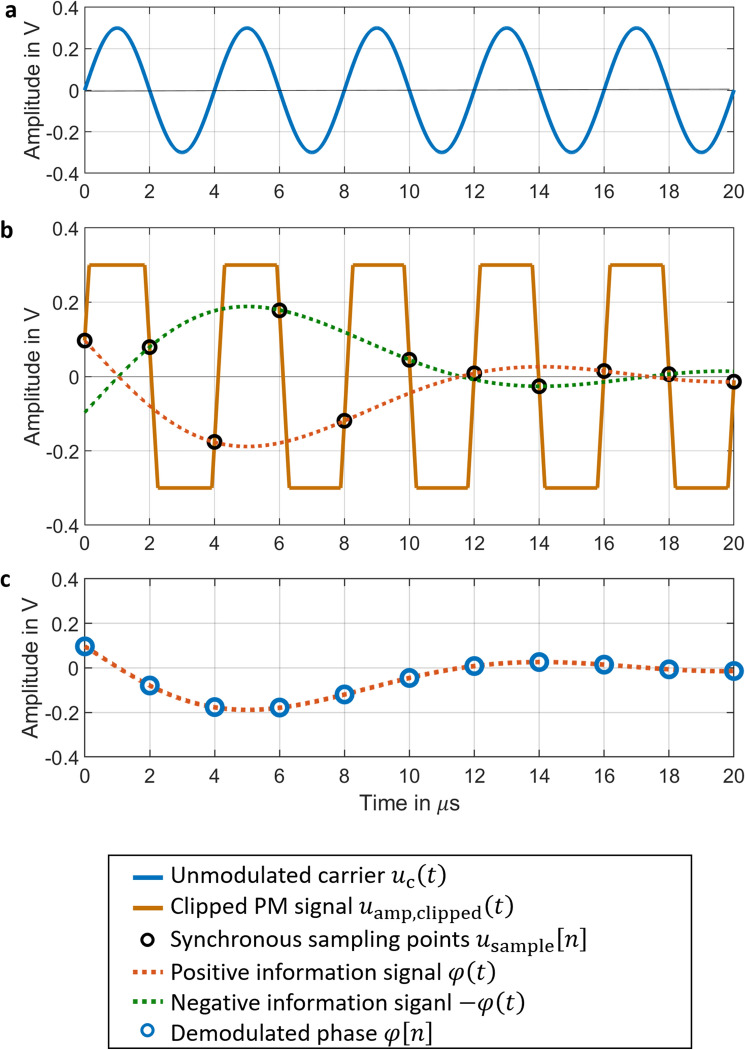
Simulation results of the synchronous sampling of a clipped phase-modulated (PM) signal. The PM signal is amplified and clipped to $u_{{{\text{amp}},{\text{ clipped}}}} \left( t \right)$ and then synchronously sampled. The PM signal with carrier amplitude $U_{{\text{c}}}$ = 0.3 V is amplified to $U_{{{\text{amp}}}} =$ 1 V and then clipped at 0.3 V. The clipping of the signal ensures that the input range $\pm K$ of the ADC remains unchanged. The sampling points at $t\left[ n \right]$ with the values $u_{{{\text{sample}}}} \left[ n \right]$ are synchronously located at the time points of the zero crossings of the unmodulated carrier $u_{{\text{c}}} \left( t \right)$ with sampling frequency $f_{{\text{s}}} = 2f_{{\text{c}}}$. The sampling values $u_{{{\text{sample}}}} \left[ n \right]$ show a linear relationship $u_{{{\text{sample}}}} \left[ n \right] = \varphi \left[ n \right]U_{{{\text{amp}}}} \cdot \left( { - 1} \right)^{n - 1} .$ with the discrete signal $\varphi \left[ n \right]$ of the modulation signal $\varphi \left( t \right)$. The demodulated phase $\varphi \left[ n \right]$ can be obtained from $\varphi \left[ n \right] = \frac{{u_{{{\text{dem}}2}} \left[ n \right]}}{{U_{{{\text{amp}}}} }}$ with $u_{{{\text{dem}}2}} \left[ n \right] = u_{{{\text{sample}}}} \left[ n \right] \cdot \left( { - 1} \right)^{n - 1}$.

Similarly, if the synchronous sampling is done with a sampling frequency $f_{{\text{s}}} = 2f_{{\text{c}}}$, because of two zero points $t\left[ n \right]$ of the carrier per cycle in Fig. 2a, the discrete signal after synchronous sampling $u_{{{\text{dem}}2}} \left[ n \right]$ follows from the sampled clipped phase modulated carrier signal by

8 $$u_{{{\text{dem}}2}} \left[ n \right] = u_{{{\text{sample}}}} \left[ n \right]\left( { - 1} \right)^{n - 1} .$$

Figure 2b shows $u_{{{\text{sample}}}} \left[ n \right]$. Therefore, in Fig. 2c, we achieve the demodulation of the discrete signal $u_{{{\text{sample}}}} \left[ n \right]$ with sampling frequency $f_{{\text{s}}} = 2f_{{\text{c}}}$ by

9 $$\varphi \left[ n \right] = \frac{{u_{{{\text{dem}}2}} \left[ n \right]}}{{U_{{{\text{amp}}}} }} = \frac{{u_{{{\text{sample}}}} \left[ n \right]}}{{U_{{{\text{amp}}}} }} \cdot \left( { - 1} \right)^{n - 1} .$$

The maximum value of the voltage $u_{{{\text{sample}}}} \left[ n \right]_{{{\text{max}}}} = MU_{{{\text{amp}}}}$ must be below the clipping threshold ${ }K$ to ensure that the amplified signal $u_{{{\text{amp}}}} \left( t \right)$ at the zero crossings $t\left[ n \right]$ of the carrier $u_{{\text{c}}} \left( t \right)$ is not falsified by clipping.

### Simulations of the synchronous sampling for different sampling rates

The spectrum of a small phase-modulated signal is approximated by the carrier and the first pairs of sidebands^32^. Higher orders of the sidebands are negligible. In order to demonstrate clear sideband amplitude variations, a single-frequency modulation phase $\varphi \left(t\right)=M\mathrm{cos}\left(2\pi {f}_{\mathrm{m}}t\right)$ is assumed as an example. We focus on the impact of clipping on the amplitude of the carrier and sidebands in the baseband when sampling is set to the zero crossings of the carrier with a sampling frequency ${f}_{\mathrm{s}}=q{f}_{\mathrm{c}},$ $\left(q\in {\mathbb{N}}^{+}\right)$ and a carrier frequency ${f}_{\mathrm{c}}$.

The single-sided amplitude spectrum ${U}_{\mathrm{amp},\mathrm{clipped}}[k]$ of the clipped signal ${u}_{\mathrm{amp},\mathrm{ clipped}}\left[n\right]$ in baseband is given by the discrete Fourier transform^33^

10 $$U_{{{\text{amp}},{\text{clipped}}}} \left[ k \right] = \frac{4K}{q}\frac{{{\text{sin}}\left( {\pi d} \right)}}{{{\text{sin}}\left( {\pi /q} \right)}}{\updelta }\left[ {k - l} \right] + \frac{{U_{{{\text{amp}}}} }}{D}{\updelta }\left[ {k - \left( {l + m} \right)} \right] + \frac{{U_{{{\text{amp}}}} }}{D}{\updelta }\left[ {k - \left( {l - m} \right)} \right],$$

where

$$d = \left\{ {\begin{array}{*{20}c} {\begin{array}{*{20}c} {\frac{q - 2}{{2q}}} & {{\text{for }}q} \\ \end{array} {\text{ = even}}} \\ {\begin{array}{*{20}c} {\frac{q - 1}{{2q}}} & {{\text{for }}q{\text{ = odd }}} \\ \end{array} } \\ \end{array} } \right.\quad {\text{and}}\quad D = \left\{ {\begin{array}{*{20}c} {\begin{array}{*{20}c} \frac{q}{2} & {{\text{for }}q} \\ \end{array} {\text{ = even}}} \\ {\begin{array}{*{20}c} q & {{\text{for }}q{\text{ = odd }}} \\ \end{array} } \\ \end{array} } \right..$$

We choose the integers $l=\frac{{f}_{\text{c}}N}{{f}_{\text{s}}}=\frac{N}{q}$ and $m=\frac{{f}_{\mathrm{m}}N}{{f}_{\mathrm{s}}}$ to sample the carrier frequency ${f}_{\text{c}}$ and modulation frequency ${f}_{\mathrm{m}}$ without leakage. $N$ is the number of points in the acquired time-domain signal. The harmonics due to clipping at high frequencies are ignored. There is a correspondence between the discrete frequency $f[k]$ and the index $k=0, 1, 2, \dots , \, \frac{N}{2}-1$ through the relation $f[k]=\frac{{f}_{\text{s}}k}{N}$. $\updelta [\cdot ]$ is the unit impulse for discrete arguments.

Equation (10) shows the result of the spectrum ${U}_{\mathrm{amp},\mathrm{clipped}}[k]$ of the clipped PM signal. The sidebands of the spectrum ${U}_{\mathrm{amp},\mathrm{ clipped}}[k]$ are proportional to the sidebands without clipping by a factor $\frac{2}{D}$. An even $q$ can obtain a double sideband amplitude compared to an odd $q$, as it can pick up one more zero point of the carrier per cycle than an odd $q$. The carrier amplitude of the spectrum ${U}_{\mathrm{amp},\mathrm{ clipped}}[k]$ for a clipped PM signal ${u}_{\mathrm{amp},\mathrm{ clipped}}\left[n\right]$ is only defined by the factor $q$ and the clipping threshold $K$. It is no longer dependent on the amplitude ${U}_{\mathrm{amp}}$ of the voltage signal ${u}_{\mathrm{amp}}\left(t\right)$ without clipping.

Through simulations, we further analyze the digitization of a clipped signal in frequency-domain for different sampling rates ${f}_{\mathrm{s}}=q{f}_{\mathrm{c}}$ in dependence on the different factor $q$. The simulation uses ${f}_{\mathrm{c}}= 40 \;\mathrm{MHz}$ as carrier frequency. This is a common center frequency in optical measurement, because it is a usual shift frequency for efficient Bragg cells. The modulation frequency is set to ${f}_{\mathrm{m}}= 0.5 \;\mathrm{MHz}$ and the modulation index in the simulation is set to $M =$ 0.1 rad in order to make the sideband in the experiment clearly distinguishable from the carrier. The amplitude of the voltage signal is set to ${ }U_{{{\text{amp}}}} = 1{\text{ V}}$. A clipping threshold $K = 1\;\mathrm{V}$ is used to present the unclipped signal. Clipping of the signal is achieved by a clipping threshold $K = 0.5\; \mathrm{V}$.

**Figure 3:**
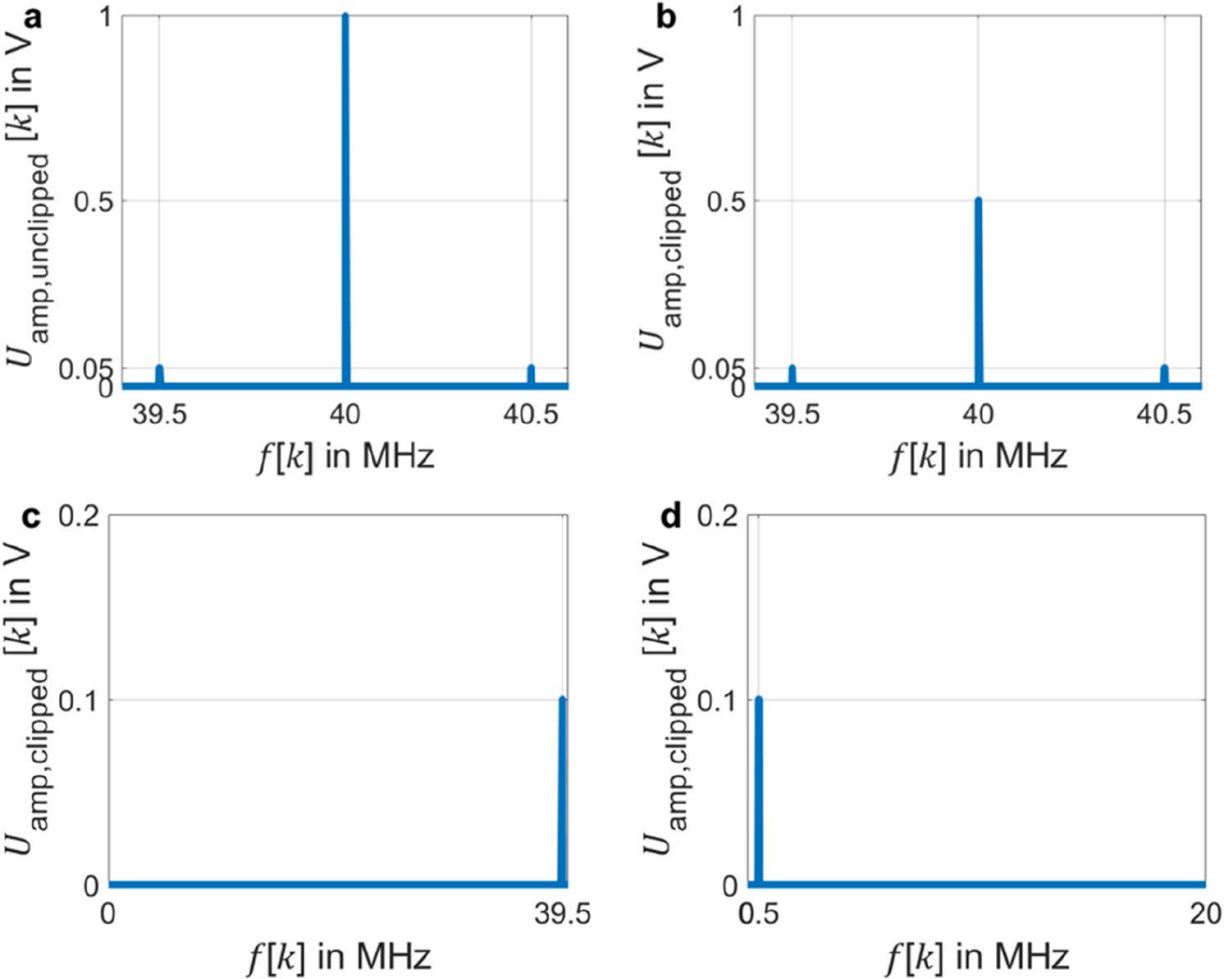
The single-sided amplitude spectrum of the simulated digital PM signal at amplitude $U_{{{\text{amp}}}} = 1{\text{ V}}$ with clipping $U_{{{\text{amp}},{\text{clipped}}}} \left[ k \right]$ by clipping threshold $K = 0.5\; {\text{V}}$ and without clipping $U_{{{\text{amp}},{\text{unclipped}}}} \left[ k \right]$ by clipping threshold $K = 1\; {\text{V}}$ at the discrete frequency $f\left[ k \right]$. (**a**) Unclipped signal sampled at sampling frequency $f_{{\text{s}}} = qf_{{\text{c}}}$ with $q = 4$. The spectrum of a small phase-modulated signal is approximated by consisting of the carrier and the first pair of sidebands. (**b**) Clipped signal sampled at sampling frequency $f_{{\text{s}}} = qf_{{\text{c}}}$ with $q = 4.{ }$ The carrier peak amplitude at 40 MHz is limited to the clipping threshold $K = 0.5 \;{\text{V}}$. The sideband peak amplitudes of 0.05 V at 39.5 MHz and 40.5 MHz are the same as for the unclipped signal. (**c**) Clipped signal sampled at sampling frequency $f_{{\text{s}}} = qf_{{\text{c}}}$ with $q = 2$. The sideband peak amplitude at 39.5 MHz becomes $M =$ 0.1 V and the carrier peak disappears. (**d**) Clipped signal sampled at sampling frequency $f_{{\text{s}}} = qf_{{\text{c}}}$ with $q = 1.$ The signal returns to the baseband and represents the modulation phase ${\varphi }\left( t \right)$ with modulation frequency $f_{{\text{m}}} = 0.5 \;{\text{MHz}}$ and modulation index $M = 0.1\; {\text{rad}}$.

Figure 3a shows the spectrum for the sampling result of an unclipped signal when the Shannon-Nyquist sampling theorem is satisfied^34^ ($q=4$). It presents the correct carrier and sideband amplitudes of the signal. As described in Eq. (5), for a digitized PM signal ${u}_{\mathrm{amp}}\left[n\right]$ without clipping, the carrier peak amplitude at 40 MHz is ${U}_{\mathrm{amp},\mathrm{unclipped}}\left[l\right]\approx {U}_{\mathrm{amp}}=1 \;\mathrm{V}$ with the small modulation index $M=0.1 \;\mathrm{rad}$. Similarly, the sideband peak amplitudes at 39.5 MHz and 40.5 MHz are ${U}_{\mathrm{amp},\mathrm{unclipped}}\left[l+m\right]={U}_{\mathrm{amp},\mathrm{unclipped}}\left[l-m\right]\approx 0.5{U}_{\mathrm{amp}}M=0.05\;\mathrm{V}$. The spectrum for the digitization of a clipped signal with a sampling rate of $q=4$ is shown in Fig. 3b. In contrast to the simulation shown in Fig. 3a, the carrier peak amplitude at 40 MHz in the spectrum of a clipped signal ${U}_{\mathrm{amp},\mathrm{clipped}}\left[n\right]$ is limited to the clipping threshold $K=0.5\; \mathrm{V}={U}_{\mathrm{amp},\mathrm{clipped}}\left[l\right]$. However, the sideband peak amplitudes of 0.05 V at 39.5 MHz and 40.5 MHz are the same as for the unclipped signal in Fig. 3a. Figure 3c depicts the spectrum for the sampling result of a clipped signal with $q=2$. Since the last frequency of the single-sideband spectrum is $\frac{{f}_{\mathrm{s}}}{2}-\frac{{f}_{\mathrm{s}}}{N}={f}_{\mathrm{c}}-RBW$ with ${f}_{\mathrm{s}}=2{f}_{\mathrm{c}}$, the carrier component at 40 MHz completely disappears. Without the anti-aliasing filter, any frequency components above $\frac{{f}_{\mathrm{s}}}{2}$ are aliased to the lower frequency interval. For the sampling rate 80 MHz, the sideband at 40.5 MHz is aliased to 39.5 MHz. Therefore, the sideband peak amplitude at 39.5 MHz becomes 0.1 V, which is equal to the sum of two sideband amplitudes ${U}_{\mathrm{amp},\mathrm{clipped}}\left[l-m\right]$ and ${U}_{\mathrm{amp},\mathrm{clipped}}\left[l+m\right]$ in Fig. 3b with $q=4$. When $q=1$, as shown in Fig. 3d, the signal returns to the baseband. The frequency and amplitude of the clipped digital PM signal for $q=1$ represent the correct modulation frequency ${f}_{\mathrm{m}}= 0.5 \; \mathrm{MHz}$ and modulation index $M=$ 0.1 rad. The results in Fig. 3c, d demonstrate that no harmonics due to clipping appear within the bandwidth of the spectrum after sampling, since the sampling points are located in the linear range of the clipped signal. For small signal modulation, this sampling result with $q = 1$ can be considered as demodulated result, while $q \ge 2$ require a more sophisticated demodulation scheme as it is shown for $q = 2$ in Eq. (9). For example, $q = 4$ would require an increase of the carrier amplitude at 40 MHz by a factor 2 before standard IQ demodulation can be applied.

### Avoiding falsifying the demodulated signal by complying limitations

In order to avoid loss of information in the PM signal, it is necessary to ensure that at least the amplitude $u_{{{\text{amp}}}} \left( {t\left[ n \right]} \right)$ at the carrier zero points $t\left[ n \right]$ is below the clipping value. Therefore, from Eq. (7), the maximum value at the carrier zero point should be

$$\left. {u_{{{\text{amp}}}} \left( {t\left[ n \right]} \right)} \right|_{{{\text{max}}}} = \varphi \left( {t\left[ n \right]} \right)_{{{\text{max}}}} U_{{{\text{amp}}}} = MU_{{{\text{amp}}}} < K.$$

However, in practice, errors in the synchronization of the ADC card to the carrier are also an important factor limiting the amplified amplitude $U_{{{\text{amp}}}}$. With defining the worst-case variation in the sampling clock as the clock jitter $t_{{\text{J}}}$, the corresponding worst-case voltage error^35^ is

$$V_{{{\text{error}}}} = \left. {\frac{{du_{{{\text{amp}},{\text{ clipped}}}} \left( t \right)}}{dt}} \right|_{{{\text{max}}}} t_{{\text{J}}} \approx \left. {\frac{{dU_{{{\text{amp}}}} {\text{sin(2}}\uppi f_{{\text{c}}} t)}}{dt}} \right|_{{{\text{max}}}} t_{{\text{J}}} = U_{{{\text{amp}}}} {2}\uppi f_{{\text{c}}} t_{{\text{J}}} < K.$$

Thus, when we combine the effects of amplified amplitude $U_{{{\text{amp}}}}$, the input range $K$ of the ADC, modulation index $M$ and clock jitter $t_{{\text{J}}}$, we can obtain a limit

11 $$M_{{{\text{max}}}} = \frac{K}{{U_{{{\text{amp}}}} }} - t_{{\text{J}}} 2\pi {\text{f}}_{{\text{c}}}$$

for the maximum modulation index $M_{{{\text{max}}}}$ for a PM signal with a small modulation index, that can still be demodulated correctly. The better synchronization between the sampling clock and the carrier signal achieve the lower the noise floor due to digitization. Thus, our method allows to transfer the requirement for bit resolution to a synchronization requirement of clocks in order to achieve maximal resolution. Synchronization of clocks is much better achievable.

## Experimental results

Based on the theoretical analysis and simulation, we use the signal generator in the first part of the experimental section to generate a PM signal. To verify the improvement by clipping of the resolution of the demodulated phase $\varphi \left[n\right]$, we show the demodulation results of the clipped and unclipped signals at synchronous sampling rates with $q=1$ and $q=2$. It is finally demonstrated that clipping can obtain a demodulation result with reduced digital noise when using the sampling method in this paper.

In the second part of the experimental section, we use this sampling method to improve the resolution of HI measurement results. It is shown as an example of a practical application of our sampling and demodulation method.

1. Experimental PM signal directly modulated by the signal generator

**Figure 4:**
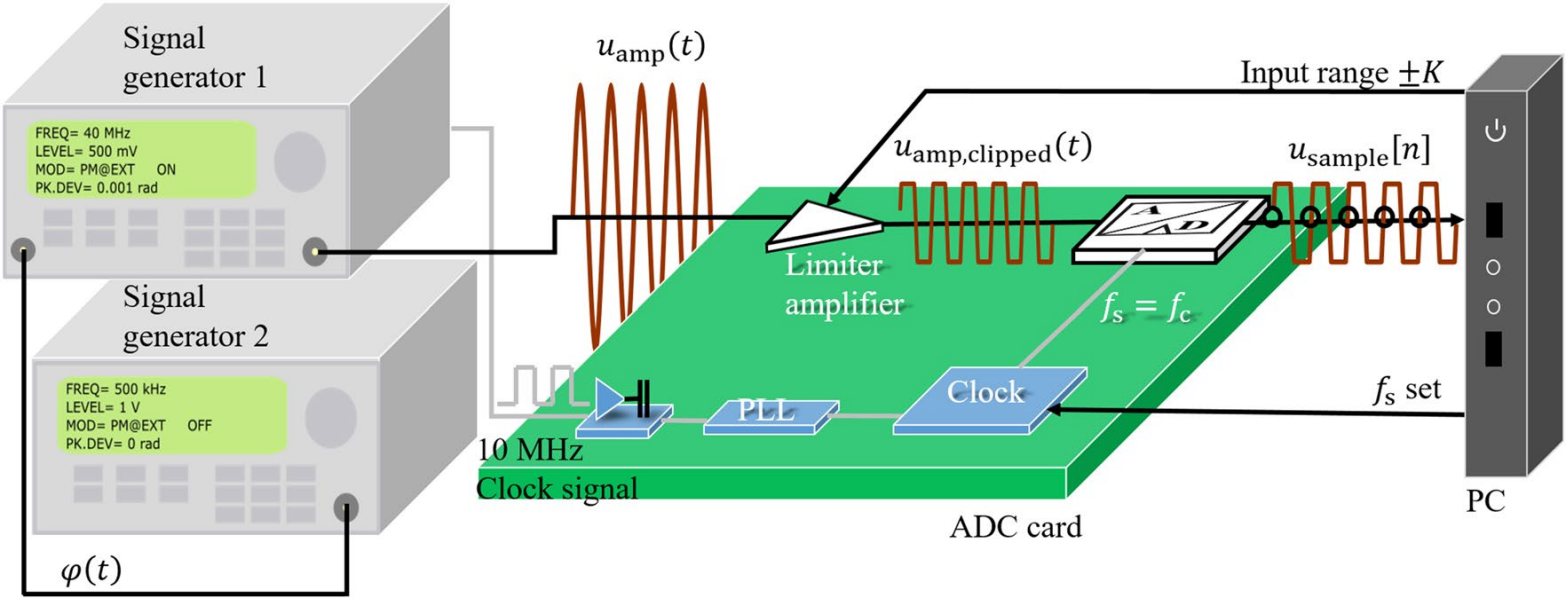
Phase modulated signal clipping and acquisition experimental setup. The carrier $u_{{\text{c}}} \left( t \right)$ with carrier frequency $f_{c}$ generated by the signal generator 1 is phase modulated by the small information signal $\varphi \left( t \right)$ generated by the signal generator 2 into $u_{{{\text{amp}}}} \left( t \right)$. The phase modulated signal $u_{{{\text{amp}}}} \left( t \right)$ is clipped by a limiter amplifier in the ADC card into $u_{{{\text{amp}},{\text{ clipped}}}} \left( t \right)$ to fit the input range $\pm K$ of the ADC. The synchronous sampling points are located at the unmodulated carrier $u_{{\text{c}}} \left( t \right)$ zero points by an internal 10 MHz Transistor–transistor logic (TTL) signal from the signal generator 1. The TTL clock signal is used to generate the sampling clock $f_{s}$ through the integrated phase-locked loop (PLL) of the ADC. The digital signal $u_{{{\text{sample}}}} \left[ n \right]$ obtained after digitization is input to the computer and analyzed. The input range and sampling rate of the ADC are controlled by the computer.

The arrangement of the experiment is illustrated in Fig. 4. It consists of two signal generators, an ADC and a computer. Signal generator 1 (Aim-TTi TGR 2050) is responsible for generating a sine carrier signal with the carrier frequency ${f}_{\mathrm{c}}=40 \; \mathrm{MHz}$. It will be phase modulated with a modulation frequency ${f}_{\mathrm{m}}= 500$ kHz provided by the signal generator 2 (M&R Systems WG1220). The modulation index is $M=0.001$ rad in order to meet the small phase modulation. The PM signal ${u}_{\mathrm{amp}}\left(t\right)$ output from signal generator 1 then is digitized by the ADC (SPECTRUM Instrumentation M4i.4421- × 8). The built-in limiter amplifier of the ADC with an amplification factor 1 allows the input range to be set by the computer. The part of the signal that exceeds the input range $K$ will be clipped. The amplitude of the voltage signal ${u}_{\mathrm{amp}}\left(t\right)$ from signal generator 1 is set to ${U}_{\mathrm{amp}}=500 \;\mathrm{mV}$. The input range of $K=200 \; \mathrm{mV}$ is used to obtain a clipped signal ${u}_{\mathrm{amp},\mathrm{ clipped}}\left(t\right)$. In order to maintain an unclipped signal ${u}_{\mathrm{amp}}\left(t\right)$, the input range is extended to $K=500 \; \mathrm{mV}$. The power of the uniformly distributed quantization noise ${P}_{\mathrm{qn}}=\frac{{LSB}^{2}}{12}$ of the $X$-bit ADC depends on the least-significant-bit $LSB=\frac{2K}{{2}^{X}}$. It means that the quantization noise power ${P}_{\mathrm{qn}}$ will decrease by a smaller $LSB$, when choosing a smaller input range $K$.

For an accurate sampling at the zero crossings of the carrier, the clock of the ADC and the signal generator 1 need to be synchronized accurately. Therefore, signal generator 1 provides an internal 10 MHz Transistor–transistor logic (TTL) signal. The TTL clock signal is used to generate the sampling clock through the integrated phase-locked loop (PLL) of the ADC. The sampling rate ${f}_{s}=q{f}_{c}$ is given by the computer. The ADC used in the experiments can achieve a maximum resolution of 16 bits. For this high digital resolution, the quantization noise is lower than the noise floor of the PM signal ${u}_{\mathrm{amp}}\left(t\right)$ from the signal generator 1 for this setup. Thus, we use the 16 bit signal as reference signal. In addition, the ADC offers a low resolution option of 8 bits, where the quantization noise will degrade the CNR of ${u}_{\mathrm{amp}}\left(t\right)$. The 8 bits resolution is chosen in order to show that the ADC resolution can be improved by clipping and proper sampling synchronization.

Before starting the synchronous sampling, the delay of the signal was checked, in order to ensure that the initial sampling point is located exactly at the zero point of the rising edge of the unmodulated carrier ${u}_{\mathrm{c}}\left(t\right)$, i.e. ${u}_{\mathrm{amp}}\left(t\right)$ with $M=0$. After the adjustment with 16-bit resolution and $q=1$, the initial sampling points of the clipped and unclipped unmodulated carrier signals fall on the zero crossings of the rising edge of the unmodulated carrier ${u}_{\mathrm{c}}\left(t\right)$ as shown in Fig. 1.

This paper proposes a simple demodulation idea for PM signals with small modulation index $M$. The carrier frequency ${f}_{\mathrm{c}}$ is used as the sampling rate ${f}_{\mathrm{s}}$ by $q=1$. The sampling points are always located at the carrier zero points with the help of clock synchronization. The acquired digital signal or ${u}_{\mathrm{sample}}[n]$ will as described in Eq. (7) directly reflect the demodulated phase $\varphi \left[n\right]$. The demodulation method with $q=1$ will simplify the digital demodulation process substantially. The amplitude ${U}_{\mathrm{amp}}$ of the voltage PM signal ${u}_{\mathrm{amp}}\left(t\right)$ without clipping is set to ${U}_{\mathrm{amp}}=500 \; \mathrm{mV}$.

**Figure 5:**
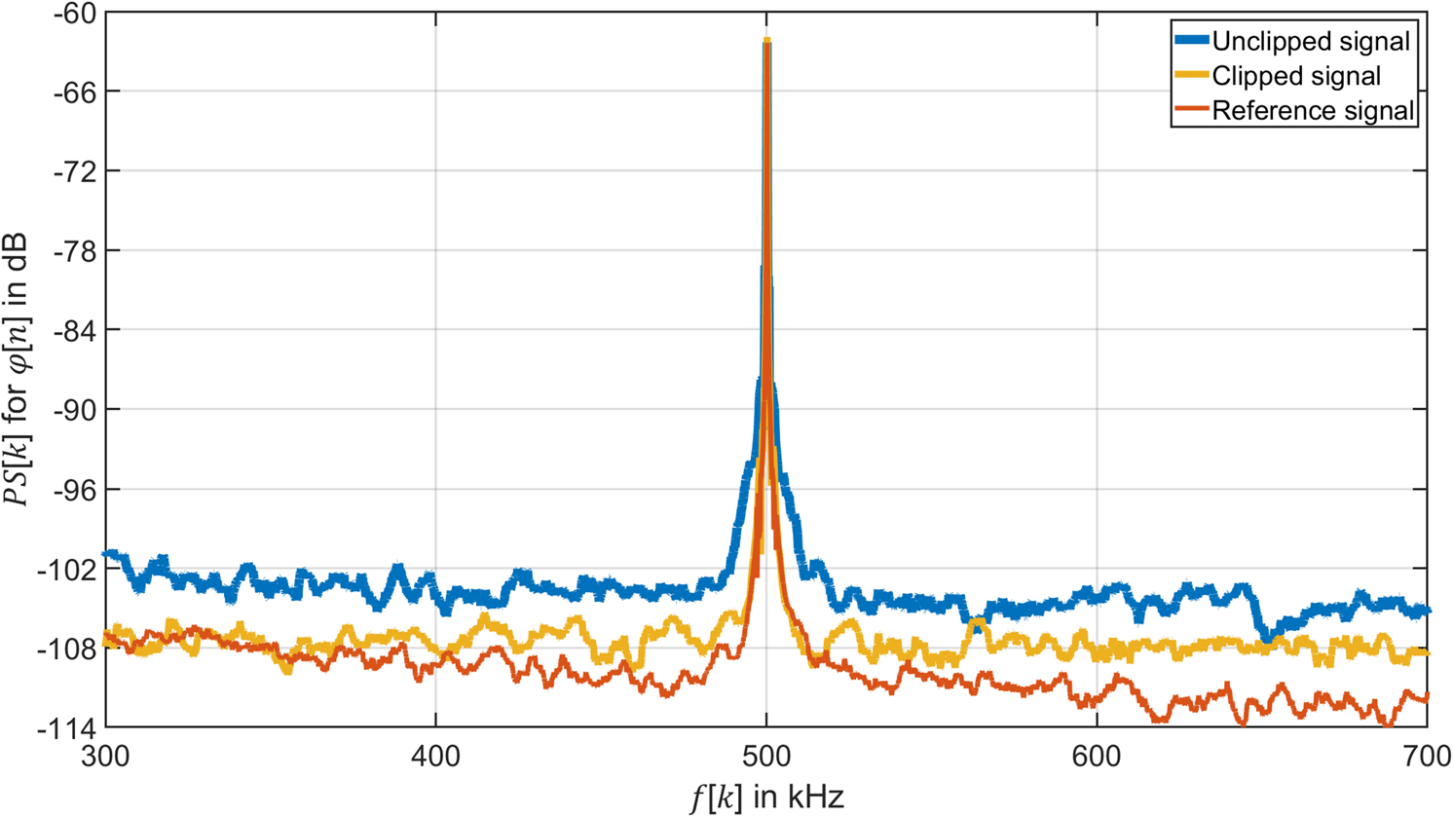
Comparison of the power spectrum $PS\left[ k \right]$ for $RBW =$ 100 Hz of the demodulated phase $\varphi \left[ n \right]$ of the clipped, unclipped and reference signal. Unclipped signal: Voltage signal $u_{{{\text{amp}}}} \left[ t \right]$ with amplitude $U_{{{\text{amp}}}} = 500\; {\text{mV}}$ sampled by an 8 bits ADC with input range $K = 500 \;{\text{mV}}$ and demodulated by the synchronous sampling with $q = 1$. Clipped signal: Voltage signal $u_{{{\text{amp}}}} \left[ t \right]$ with amplitude $U_{{{\text{amp}}}} = 500\; {\text{mV}}$ sampled by an 8 bits ADC with input range $K = 200 \;{\text{mV}}$ and demodulated by the synchronous sampling with $q = 1$. Reference signal: Voltage signal $u_{{{\text{amp}}}} \left[ t \right]$ with amplitude $U_{{{\text{amp}}}} = 500 \;{\text{mV}}$ sampled by a 16 bits ADC with input range $K = 500 \;{\text{mV}}$ and demodulated by the IQ demodulation with $q = 4$.

In order to observe the effect of clipping on the quantization noise power ${P}_{\mathrm{qn}}$ of the demodulated phase $\varphi \left[n\right]$, the power spectrum $PS\left[k\right]$ of the demodulated phase $\varphi \left[ n \right]$ in $RBW =$ 100 Hz

12 $$\left. {PS\left[ k \right]} \right|_{{{\text{dB}}}} = 10\log_{10} \left( {\frac{{\Phi \left[ k \right]^{2} }}{{2 \cdot 1\; {\text{rad}}^{2} }}} \right)$$

is given by the amplitude spectrum $\Phi [k]$ of the demodulated phase $\varphi \left[n\right]$ at frequency $f[k]$. The power spectrum $PS\left[k\right]$ of the demodulated phase $\varphi \left[n\right]$ at frequency $f[k]$ is shown in Fig. 5 for the three cases reference signal, unclipped signal, and clipped signal when $q=1$ is used.

Figure 5 shows three curves, the spectrum of demodulated unclipped signal, clipped signal and reference signal. The unclipped signal and clipped signal are sampled by an 8-bit ADC and then demodulated by the demodulation method with $q=1$. The reference signal is sampled by a 16-bit ADC and demodulated by the IQ demodulation with $q = 4$. The three curves demonstrate the same amplitude and frequency in the power spectrum $PS\left[ k \right]$ of the demodulated phase $\varphi \left[ n \right]$. It illustrates that the demodulation method with $q = 1$ can obtain the same demodulated phase $\varphi \left[ n \right]$ as the IQ demodulation. For the unclipped signal (blue curve), the resolution of the demodulated phase $\varphi \left[ n \right]$ is limited by the quantization noise of the ADC. The clipping can improve the SNR of the demodulated phase ${ }\varphi \left[ n \right]$, as shown as the orange curve. As the input range is set to a smaller value, i.e. $K = \pm 200\; {\text{mV}}$, the quantization noise power is dropped. The SNR of the clipped signal as shown in Fig. 5 is approximately 3 dB higher than that of the unclipped signal.

The spectral aliasing effect occurs when using a sampling rate of $f_{{\text{s}}} = f_{{\text{c}}} =$ 40 MHz below 2 $f_{{\text{c}}}$ and without an anti-aliasing filter. The frequency components in the spectral interval higher than $\frac{{f_{{\text{s}}} }}{2} =$ 20 MHz will be loaded on the frequency components lower than 20 MHz. As a result, the sideband components appear back to the baseband by the effect of undersampling and located at 500 kHz as shown in Fig. 3d for $q = 1$. However, the noise of the clipped signal with $q = 1$ between 0 Hz and 20 MHz increases by a factor 2 in respect to the 16 bit-reference signal with $q = 4$ and IQ modulation due to the superposition of the noise at high frequencies. Therefore, the SNR of the clipped signal with $q = 1$ (orange curve) is still about 3 dB lower compared to that of the reference signal (red curve), as shown in Fig. 5. In addition, near frequency $f\left[ k \right] = 250\; {\text{kHz}}$, the noise floor of the clipped signal with $q = 1$ gradually overlaps with the reference signal. The reason is that the carrier signal $u_{{\text{c}}} \left( t \right)$ generated by signal generator 1 (Fig. 4) carries the phase noise. The phase noise near the carrier acts gradually as the most dominant noise component and covers the digitization noise.

**Figure 6:**
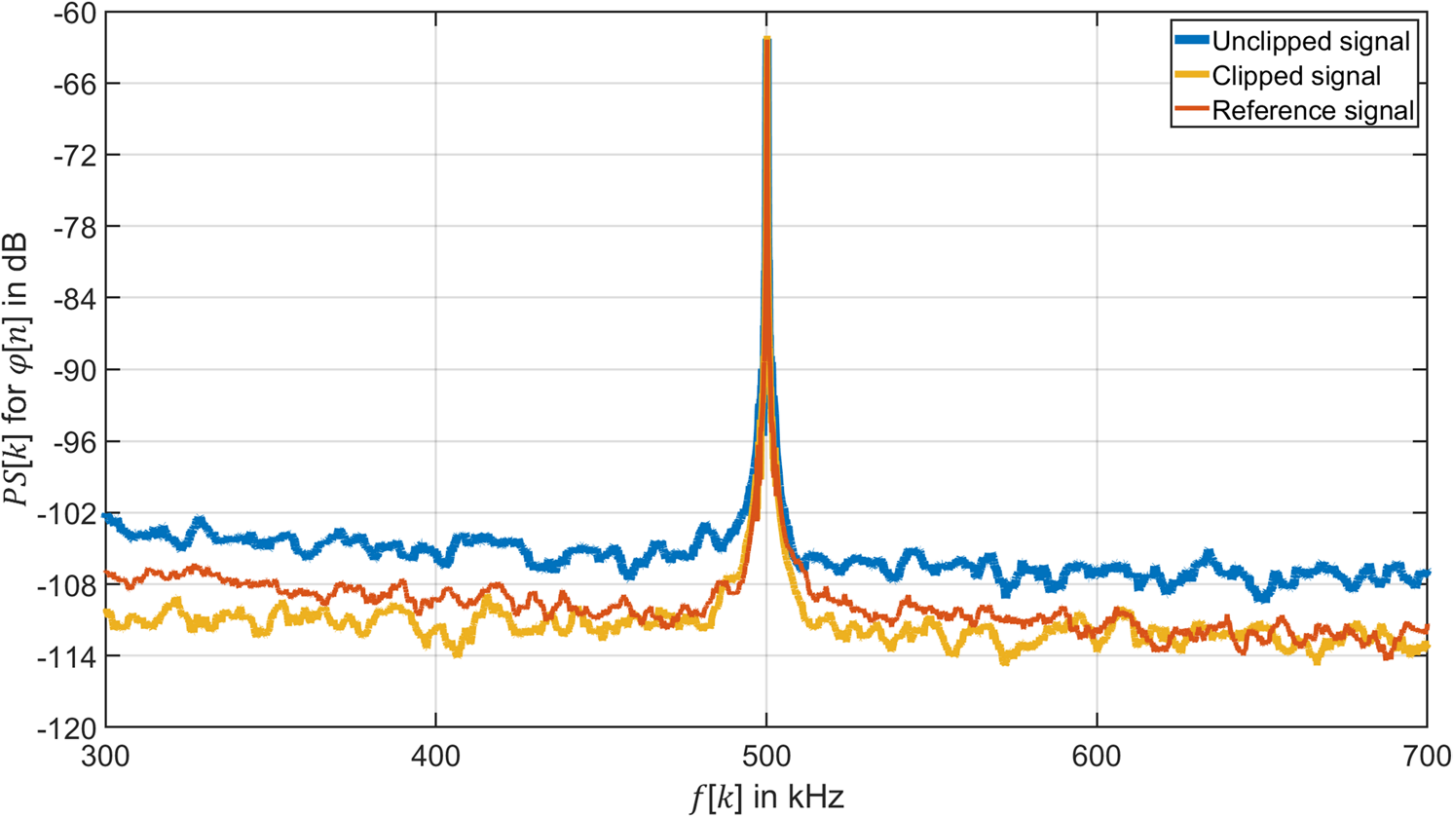
Comparison of the power spectrum $PS\left[ k \right]$ for $RBW =$ 100 Hz of the demodulated phase $\varphi \left[ n \right]$ of the clipped, unclipped and reference signal. Unclipped signal: Voltage signal $u_{{{\text{amp}}}} \left[ t \right]$ with amplitude $U_{{{\text{amp}}}} = 500 \; {\text{mV}}$ sampled by an 8 bits ADC with input range $K = 500 \;{\text{mV}}$ and demodulated by the synchronous sampling with $q = 2$. Clipped signal: Voltage signal $u_{{{\text{amp}}}} \left[ t \right]$ with amplitude $U_{{{\text{amp}}}} = 500 \;{\text{mV}}$ sampled by an 8 bits ADC with input range $K = 200 \;{\text{mV}}$ and demodulated by the synchronous sampling with $q = 2$. Reference signal: Voltage signal $u_{{{\text{amp}}}} \left[ t \right]$ with amplitude $U_{{{\text{amp}}}} = 500 \; {\text{mV}}$ sampled by a 16 bits ADC with input range $K = 500 \;{\text{mV}}$ and demodulated by the IQ demodulation with $q = 4$.

A sampling rate $f_{{\text{s}}}$ with $q = 2$ can demodulate PM signals in respect to Eq. (9). The power spectrum $PS\left[ k \right]$ of the phase $\varphi \left[ n \right]$ demodulated by the $q = 2$ algorithm is shown for three cases in Fig. 6. The three curves demonstrate the same amplitude and frequency in the power spectrum $PS\left[ k \right]$ of the demodulated phase $\varphi \left[ n \right]$. It proves that the demodulation method with $q =$ 2 can demodulate PM signals correctly for a small modulation index $M$. Figure 6 reflects that clipping compensates digital noise from the low-resolution of the ADC, so that the clipped signal (orange curve) can get a 6 dB improvement for the SNR of the demodulated phase $\varphi \left[ n \right]$ than the unclipped signal (blue curve).

In contrast to the case of ${ }q = 1$, sampling with $q = 2$ cannot transfer the signal directly to the baseband (Fig. 3c, d). However, for each carrier cycle, sampling with $q = 2$ can acquire one more sample point containing phase information compared to sampling with $q = 1$. The advantage of the demodulation by the $q = 2$ algorithm is avoiding the 3 dB reduction of the SNR of the demodulated phase $\varphi \left[ n \right]$ due to the aliasing effect. Therefore, the noise floor of the clipped signal (orange curve) is the same as that of the reference signal (red curve) in Fig. 6.

2. Experimental proof of the resolution improvement of a heterodyne interferometer

Light with the wavelength $\lambda$ reflected from an object moving with constant velocity $v$ occurs as a change in frequency ${\Delta }f = 2\frac{v}{\lambda }$ proportional to the velocity $v$ of the object (Doppler Effect). The changes in frequency are detected by an interferometer. Within the interferometer, the laser beam is divided into a reference beam and a measurement beam. The electric field of the measurement beam is phase-modulated by the modulation phase $\varphi \left( t \right) = \frac{2\pi }{\lambda }OPL\left( t \right)$ with the variation of the optical path length $OPL\left( t \right)$. The optical path length $OPL\left( t \right) = 2s\left( t \right)$ depends on the motion $s\left( t \right)$ of the reflective surface of the specimen with the time ${ }t$. The derivative of the displacement $s\left( t \right)$ results in the time-dependent velocity signal $v\left( t \right)$. The field of the measurement beam with the frequency $\omega_{{\text{m}}}$ interferes with the reference field with the frequency $\omega_{{\text{r}}}$ and is then converted by a balanced photodetector to the current signal $i_{{\text{s}}} \left( {\text{t}} \right).$ The current signal

13 $$i_{{\text{s}}} \left( {\text{t}} \right) = 2k\varepsilon \sqrt {P_{{\text{m}}} P_{{\text{r}}} } \sin \left( {\omega_{{\text{m}}} t - \omega_{{\text{r}}} t + \varphi \left( t \right)} \right) = I_{s} \sin \left( {\omega_{{\text{m}}} t - \omega_{{\text{r}}} t + \varphi \left( t \right)} \right)$$

recorded by balanced photodiodes contains a contribution depending on the displacement $s\left( t \right)$ of the object through the modulation phase $\varphi \left(t\right)=\frac{4\pi }{\lambda }s\left(t\right)$. The parameter $k$ in unit of A/W represents the responsivity of the photodiode. The factor $\varepsilon$ is the heterodyne efficiency (matched wave fronts for the interfering beams). When an acousto-optic modulator shifts the frequency of a measured beam by ${\omega }_{\mathrm{c}}$ relative to the frequency of a reference beam, the resulting frequency difference between the two beams is ${\omega }_{\mathrm{m}}-{\omega }_{\mathrm{r}}={\omega }_{\mathrm{c}}$. The current signal can be expressed as ${i}_{\mathrm{s}}\left({\text{t}}\right)=2k\varepsilon \sqrt{{P}_{\mathrm{m}}{P}_{\mathrm{r}}} \mathrm{sin}\left({\omega }_{\mathrm{c}}t+\varphi \left(t\right)\right)$. The detection of the difference in optical path is called a heterodyne interferometry (HI). In the field of optical metrology, HI^36,37^ can provide a non-contact, high-resolution dynamic analysis of displacement amplitudes. Small vibration amplitudes and high frequencies occur when testing of microelectromechanical systems^7^.

However, the quantum character of light^38^ will produce a photon shot-noise power and consequently cause fluctuations of the photocurrent ${i}_{\mathrm{s}}\left({\text{t}}\right)$. The theoretical carrier-to-noise ratio (CNR) in decibels (dB) at the photodetector can be estimated by ${CNR}_{\mathrm{shot\;noise}, \mathrm{dB}}=10{\mathrm{log}}_{10}\frac{{\eta \cdot {\varepsilon }^{2}\cdot P}_{\mathrm{m}}{P}_{\mathrm{r}}}{h\cdot \frac{c}{\lambda }\cdot RBW\left({P}_{\mathrm{m}}+{P}_{\mathrm{r}}\right)}$ ^39^ with the Planck's constant $h= 6.626\cdot {10}^{-34} \;\mathrm{ Js}$, the speed of light in air $c \approx 3\cdot {10}^{8} \;\mathrm{m}/\mathrm{s}$, the ideal heterodyne efficiency $\varepsilon =1$ and the perfect reflectivity $\eta =1$. It will reach ${CNR}_{\mathrm{shot\;noise}, \mathrm{dB}}=162 \;\mathrm{ dB}$ in resolution bandwidth (RBW) $RBW=1 \; \mathrm{Hz}$ when using a wavelength $\lambda$=1550 nm, a power of the measuring light ${P}_{\mathrm{m}}=5 \;\mathrm{mW}$ and a power of reference light ${P}_{\mathrm{r}}=5 \; \mathrm{mW}$^40^. The resolution limit ${s}_{\mathrm{rl}}$ for the demodulated displacement $s[n]$ $\left(n\in {\mathbb{N}}^{+}\right)$ based on the CNR limited by photon shot noise should reach^40^

14 $$s_{{{\text{rl}}}} = \frac{{\lambda {\Delta }\varphi }}{{4 \cdot {\uppi }}} \approx \frac{{2{\text{ fm}}}}{{\sqrt {{\text{Hz}}} }} {\text{with}} {\Delta }\varphi = \frac{\sqrt 2 }{{\sqrt {CNR} }}$$

with $$CNR={CNR}_{\mathrm{shot\;noise}}$$.

**Figure 7:**
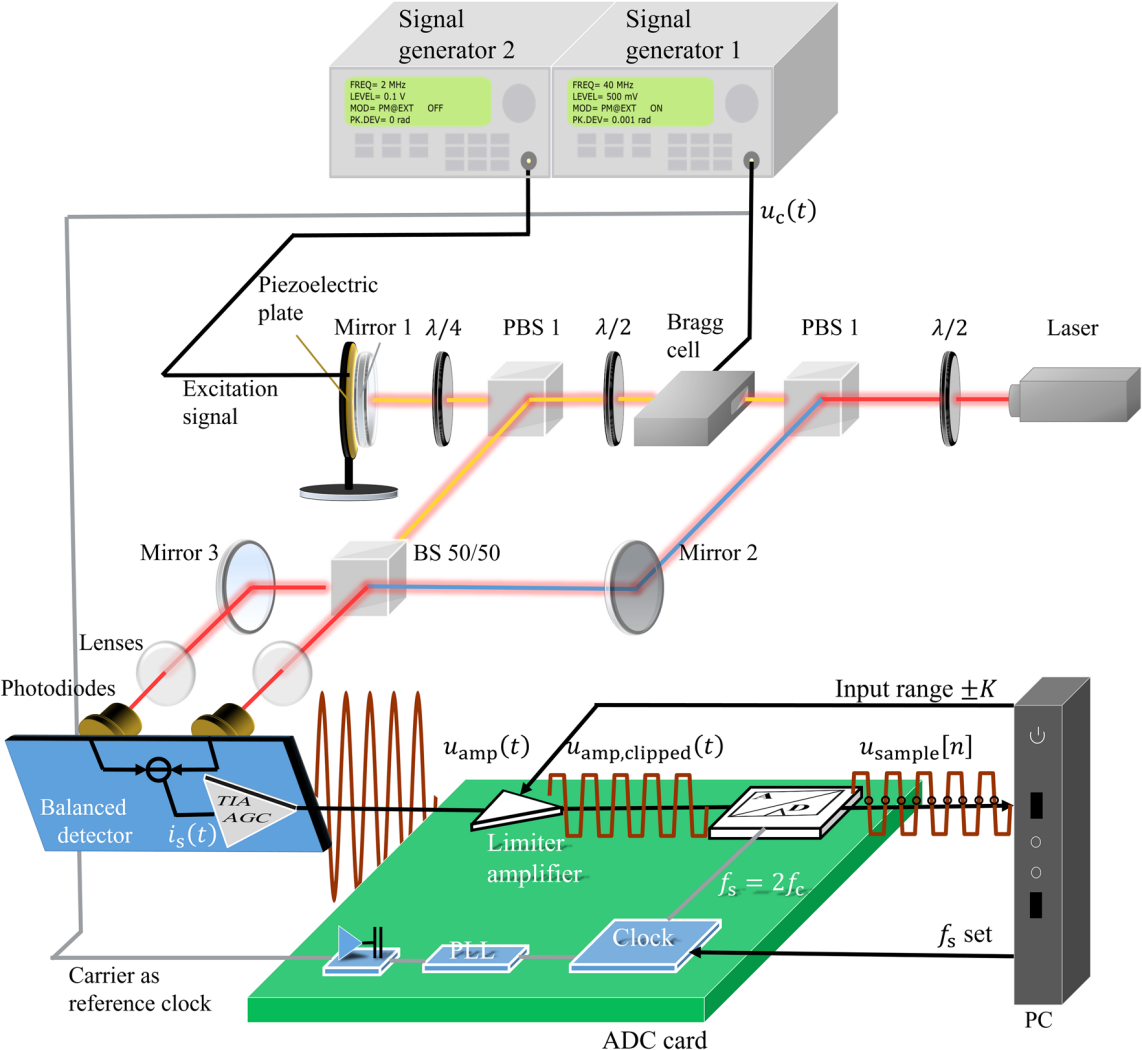
Heterodyne interferometer (HI) signal acquisition and clipping experimental setup. The laser beam is divided into a measurement beam (orange beam) and a reference beam (blue beam). The measurement beam (orange beam) produces a frequency shift $f_{{\text{c}}}$ at the Bragg cell and illuminates the surface of the target object. The frequency shift $f_{{\text{c}}}$ as carrier frequency depends on the carrier signal $u_{{\text{c}}} \left( t \right)$ generated by signal generator 1. The target object is a mirror with a piezoelectric plate. The piezoelectric plate vibrates with the excitation signal generated by the signal generator 2. The measurement beam is reflected on the surface of the object and interferes with the reference beam at the photodiode. The balanced detector produces a phase modulated photocurrent signal $i_{{\text{s}}} \left( {t} \right)$. The phase-modulated photocurrent signal $i_{{\text{s}}} \left( {t} \right)$ is converted into a phase-modulated voltage signal $u_{{{\text{amp}}}} \left( t \right)$ with a suitable amplitude of $U_{{{\text{amp}}}}$ by a transimpedance amplifier (TIA) with AGC. The phase modulated signal $u_{{{\text{amp}}}} \left( t \right)$ is clipped by limiter amplifier in the ADC card into $u_{{{\text{amp}},{\text{ clipped}}}} \left( t \right)$ to fit the input range $\pm K$ of the ADC. The unmodulated carrier signal $u_{{\text{c}}} \left( t \right)$ is used to generate the sampling clock $f_{{\text{s}}} = 2f_{{\text{c}}}$ through the integrated phase-locked loop PLL of the ADC. The digital signal $u_{{{\text{sample}}}} \left[ n \right]$ obtained after digitization is input to the computer and analyzed.

In the signal chain as shown in Fig. 7, the transimpedance amplifier (TIA) amplifies and converts the current signal ${i}_{\mathrm{s}}\left(t\right)$ to the voltage signal ${u}_{\mathrm{amp}}\left(t\right)$.^41^ The automatic gain control (AGC) is a closed-loop feedback regulation circuit in an amplifier^42^. It purposes to maintain the proper signal amplitude ${U}_{\mathrm{amp}}=500 \;\mathrm{mV}$ of the voltage signal ${u}_{\mathrm{amp}}\left(t\right)$ at its output despite changes in signal amplitude at the input. The CNR of the voltage signal ${u}_{\mathrm{amp}}\left(t\right)$ is measured by a spectrum analyzer (HP 8591E) as 144 dB. The demodulated displacement based on this CNR can reach 11 $\mathrm{fm}/\sqrt{\mathrm{Hz}}$. The subsequent digitization process of the analog-to-digital converter (ADC) may introduce quantization noise on the digital signal ${u}_{\mathrm{amp}}[n]$^31^. The theoretical CNR in dB for an *X*-bit ADC with a sampling rate ${f}_{\mathrm{s}}$ for RBW 1 Hz can be denoted as ${CNR}_{\mathrm{qn}, \mathrm{dB}}=6.02\cdot X+1.76+10\log\frac{{f}_{\mathrm{s}}}{2}$^31^. For example, an 8-bit ADC with a sampling rate ${f}_{\mathrm{s}}=250 \;\text{mega samples per second} (\mathrm{MSps})$ can only achieve the CNR limited by quantization noise of $131\;\mathrm{ dB}$ in a RBW of 1 Hz. Therefore, quantization noise is the dominating noise component in this signal chain. The resolution limit ${s}_{\mathrm{rl}}$ for the demodulated displacement $s[n]$ based on the CNR limited by quantization noise can reach only $50\;\mathrm{ fm}/\sqrt{\mathrm{Hz}}$ for our setup if we do not employ our synchronous sampling and clipping method.

In order to break the digital-noise limitation of the ADC on the resolution limit ${s}_{\mathrm{rl}}$ of the demodulated displacement $s[n]$, we demonstrate the experiment results of our sampling and processing method for a clipped PM signal with small modulation index $M\ll 1$ in the application of a HI. For the HI, the displacement amplitude $\widehat{s}$ and the wavelength $\lambda$ of the measure light define the modulation index $M=\frac{4\pi }{\lambda }\widehat{s}$.

The setup of the experiment of HI is illustrated in Fig. 7. It consists of the HI optical setup part, the balance detector, the ADC card and the PC. In the HI optical part, the laser beam is divided into a measurement beam and a reference beam. The measurement beam produces a frequency shift ${f}_{\mathrm{c}}$ at the Bragg cell and illuminates the surface of the target object. The frequency shift ${f}_{\mathrm{c}}$ depends on the excitation signal ${u}_{\mathrm{c}}(t)$ generated by signal generator 1. The target object is a mirror with a piezoelectric plate. The piezoelectric plate vibrates with the input voltage generated by the signal generator 2. As a result, a corresponding displacement change $s(t)$ of the mirror surface is generated and translated into the optical path length difference of the measurement beam. The measurement beam is reflected on the surface of the object and interferes with the reference beam at the photodiode to produce a phase modulated photodetector signal with modulation signal $\varphi \left(t\right)=\frac{4\pi }{\lambda }s(t)$ and amplitude ${I}_{\mathrm{s}}=2k\varepsilon \sqrt{{P}_{\mathrm{m}}{P}_{\mathrm{r}}}=10 \;\mathrm{mA}$ dependent on the responsivity of the photodiode $k=1 \;\mathrm{A}/\mathrm{W}$, the ideal heterodyne efficiency $\varepsilon =1$, the power of the measurement beam ${P}_{\mathrm{m}}=5\; \mathrm{mW}$ and reference beam ${P}_{\mathrm{r}}=5 \;\mathrm{mW}$. Signal generator 1 (Aim-TTi TGR 2050) is responsible for generating a sine carrier signal with the carrier frequency ${f}_{\mathrm{c}}=40 \;\mathrm{MHz}$. Signal generator 2 (M&R Systems WG1220) generates a 2 MHz sinusoidal voltage signal with an amplitude of 0.1 V to excite a vibration of the piezoelectric plate. Because the amplitude of the piezoelectric plate is tiny, it satisfies our condition of small phase modulation. A mirror is glued on the piezoelectric plate to ensure high reflectivity of the specimen surface and avoids a limitation by photon shot noise.

The phase-modulated photocurrent signal ${i}_{\mathrm{s}}\left({t}\right)$ is converted into a phase-modulated voltage signal ${u}_{\mathrm{amp}}\left(t\right)$ with a suitable amplitude of ${U}_{\mathrm{amp}}=500 \;\mathrm{mV}$ by a TIA with AGC. The sampling and digitizing process for the amplified PM voltage signal is the same as in the first experiment. The PM signal ${u}_{\mathrm{amp}}\left(t\right)$ is digitized by the ADC (SPECTRUM Instrumentation M4i.4421- × 8). The built-in limiter amplifier of the ADC with an amplification factor 1 allows the input range to be set by the computer. The input range of $K=200 \;\mathrm{mV}$ is used to obtain a clipped signal ${u}_{\mathrm{amp},\mathrm{ clipped}}\left(t\right)$ and the input range of $K=500 \;\mathrm{mV}$ is used to obtain an unclipped signal ${u}_{\mathrm{amp}}\left(t\right)$. The slew rate limits the amplitude ${U}_{\mathrm{amp}}$ of the carrier to $\frac{\mathrm{slew\;rate}}{2\pi f}$. The transimpedance amplifier OPA690 has a slew rate of $1800\; \mathrm{V}/\mathrm{\mu s}$ and the 40 MHz signal can be amplified to 7 V. The limiting amplifier in the ADC (SPA.1411) has a slew rate of more than $2500\; \mathrm{V}/\mathrm{\mu s}$, ensuring that a $f=$ 40 MHz signal can be amplified to 10 V. In our experiments, we use a signal amplitude of ${U}_{\mathrm{amp}}=$ 500 mV. The carrier signal is divided into two identical signals, one is used to excite the Bragg cell to generate the carrier, and the other is used to generate the sampling clock through the integrated phase-locked loop (PLL) of the ADC. In this experiment, we set the sampling rate ${f}_{\mathrm{s}}={2f}_{\mathrm{c}}$ because we proved in the first experiment that sampling with $q=2$ has better results. The ADC was set to 8 bit mode.

**Figure 8:**
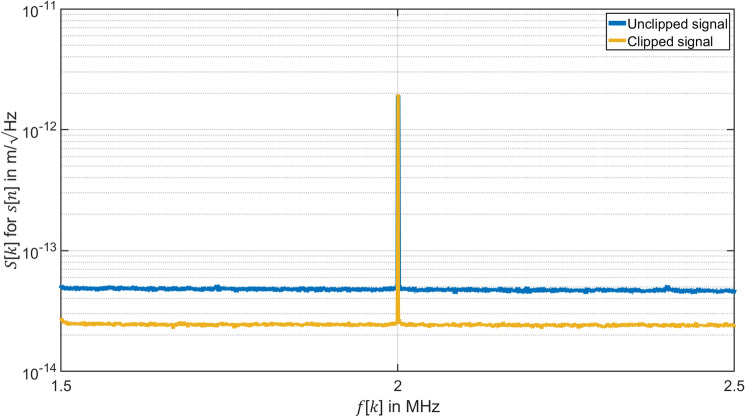
Comparison of the resolution limit by spectrum $S\left[ k \right]$ of the displacement $s\left[ n \right]$ measured by HI with $RBW =$ 1 Hz of the clipped and unclipped signal when using the demodulation method with $q = 2$. Unclipped signal: Voltage signal $u_{{{\text{amp}}}} \left( t \right)$ measured by HI with amplitude $U_{{{\text{amp}}}} = 500 \;{\text{mV}}$ sampled by an 8-bit ADC with input range $K = 500\; {\text{mV}}$ and demodulated by the demodulation method for sampling frequency $f_{{\text{s}}} = 250 \;{\text{MHz}}$. Clipped signal: Voltage signal $u_{{{\text{amp}}}} \left( t \right)$ measured by HI with amplitude $U_{{{\text{amp}}}} = 500\; {\text{mV}}$ sampled by an 8-bit ADC with input range $K = 200 \;{\text{mV}}$ and demodulated by the demodulation method for sampling frequency $f_{{\text{s}}} = 80 \;{\text{MHz}}$.

Equation (9) allows to demodulate PM signals if a sampling rate ${f}_{\mathrm{s}}=q {f}_{\mathrm{c}}$ with $q=2$ is selected. The equation $s\left[n\right]=\frac{\lambda }{4\pi }\varphi \left[n\right]$ yields the displacement of the specimen. Figure 8 shows the spectrum $S[k]$ of the displacement $s\left[n\right]$. The two curves demonstrate the same amplitude and frequency in the spectrum $S\left[k\right]$ of the displacement $s\left[n\right]$. However, clipping compensates digital noise from the low-resolution of the ADC, so that the clipped signal (orange curve) can get a better level for the SNR of the demodulated displacement $s\left[n\right]$ than the unclipped signal (blue curve). It improves the resolution limit ${s}_{\mathrm{rl}}$ of our HI measurement from $50\;\mathrm{ fm}/\sqrt{\mathrm{Hz}}$ limited by digital noise to $23\;\mathrm{ fm}/\sqrt{\mathrm{Hz}}$. For the resolution $23\;\mathrm{ fm}/\sqrt{\mathrm{Hz}}$, the corresponding CNR of the voltage signal ${u}_{\mathrm{amp}}\left(t\right)$ can estimated at 137.6 dB. We measured the voltage signal ${u}_{\mathrm{amp}}\left(t\right)$ by a spectrum analyzer (HP 8591E) and analyzed carrier and sidebands with different frequency bands and reference powers. We estimated a CNR of $CNR=144 \;\mathrm{dB}$. We interpret the results as follows. The jitter in the synchronization system reduces the CNR from 144 to 137.6 dB. A better synchronization between the sampling clock and the carrier signal can further improve the resolution limit.

## Discussion

**Figure 9:**
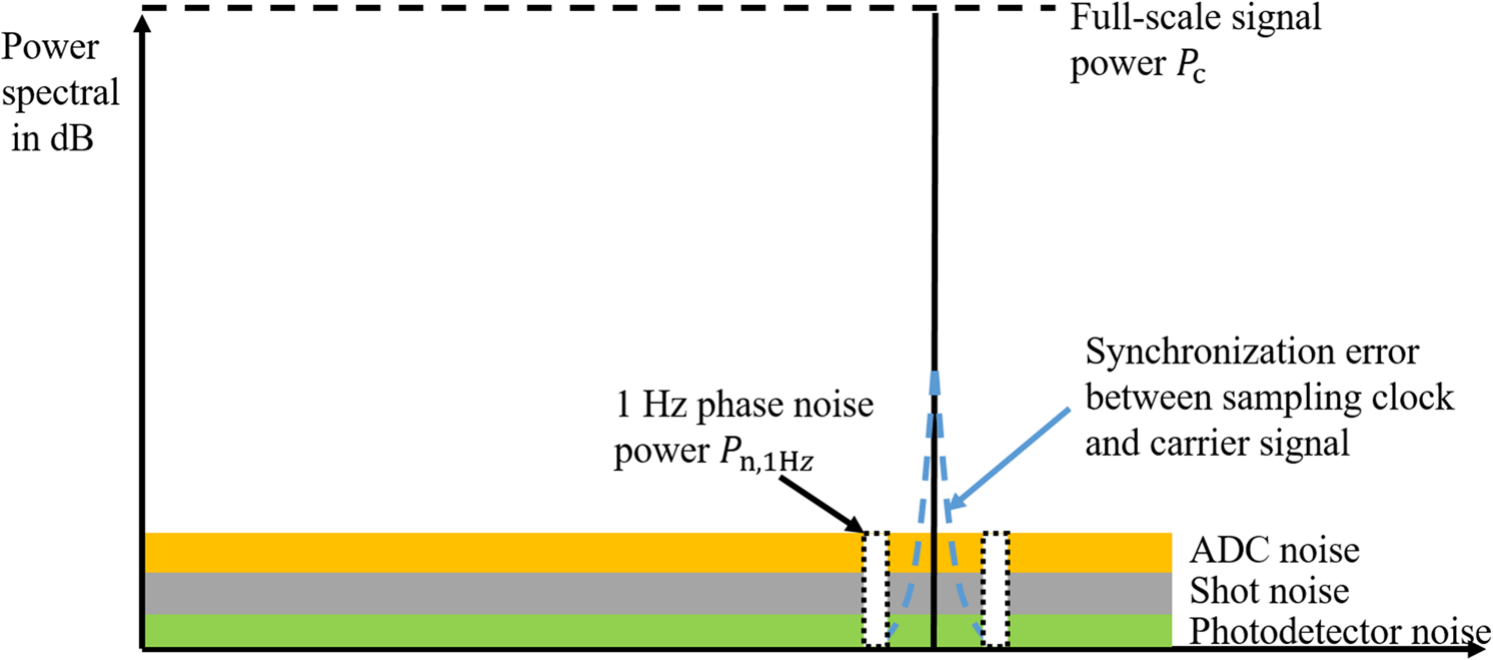
A spectrum of a full-scale carrier signal of heterodyne interferometer with noise.

The resolution of the optical signal from HI is limited only by the quantum noise (shot noise) of the light. The coherent amplification by the reference light is sufficient so that the complete detector noise (thermal noise, amplifier voltage and current noise) is below the photon shot noise as shown in Fig. 9. During the analog-to-digital conversion, errors in the synchronization of the sampling clock and the carrier lead to an increase in phase noise close to the carrier. In addition, the quantization noise of the ADC is superimposed on the signal. Here we focus only on the white noise spectrum of the ADC (consisting of quantization noise or thermal noise of the ADC), as it represents the minimum resolution achievable by the HI.

The resolution of heterodyne interferometer can be calculated by phase noise from the two sidebands. The phase noise $\Delta \varphi$ of the demodulated phase $\varphi \left[n\right]$ of HI considering the averaged noise from the upper and lower sidebands is estimated as^40^

15 $$\Delta \varphi = \frac{\sqrt 2 }{{\sqrt {CNR_{{{\text{qn}}}} } }}.$$

According to our sampling method and the demodulation method with $q=2$, we can amplify the voltage signal amplitude to ${U}_{\mathrm{amp}}\approx \frac{K}{{M}_{\mathrm{max}}}$ [Eq. (11)], when using a good synchronization (${t}_{\mathrm{J}}\ll \frac{M}{2\pi {\text{f}}_{\text{c}}}$). It can enhance the resolution $\Delta \varphi$ of the demodulated phase $\varphi \left[n\right]$ for a PM signal with a maximal possible modulation index ${M}_{\mathrm{max}}$ to

16 $$\Delta \varphi_{{{\text{new}}}} = \frac{K}{{U_{{{\text{amp}}}} }}\frac{\sqrt 2 }{{\sqrt {CNR_{{{\text{qn}}}} } }} = \frac{{\sqrt 2 M_{{{\text{max}}}} }}{{\sqrt {CNR_{{{\text{qn}}}} } }}.$$

The complete bit resolution of the ADC is used to quantize the interesting modulation signal $\varphi (t)$ with a maximum amplitude $M<\frac{\pi }{4}$ by amplification and clipping of the PM signal. In contrast, in conventional sampling techniques, the carrier amplitude is limited by the input range of the ADC (${U}_{\mathrm{amp}}=K$), which results in only partial bit resolution for small phase modulated signals.

**Figure 10:**
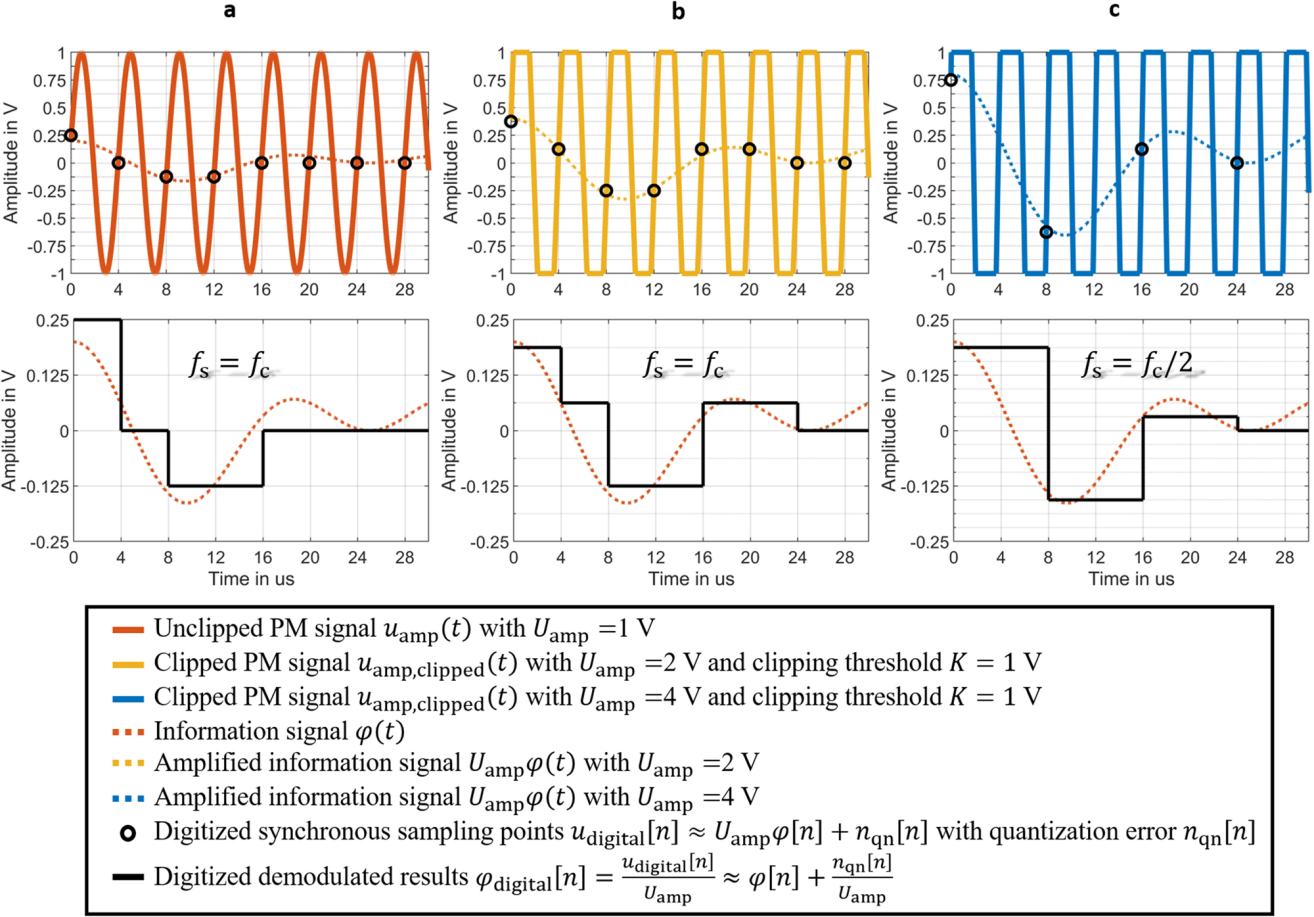
Simulation results of the digitization of the unclipped and clipped broadband, phase-modulated (PM) signals by a 4-bit ADC. The quantization error $n_{{{\text{qn}}}}$ can take a value between $\pm LSB/2$ with the least-significant-bit $LSB = 0.125\; {\text{V}}$ for a 4-bit ADC. (**a**) The PM signal is amplified to $u_{{{\text{amp}}}} \left( t \right)$ with $U_{{{\text{amp}}}} =$ 1 V without clipping and then synchronously sampled with $f_{{\text{s}}} = f_{{\text{c}}}$ and digitized to $u_{{{\text{digital}}}} \left[ n \right]$. The digitized demodulated results $\varphi_{{{\text{digital}}}} \left[ n \right]$ have the typical quantization error effect of $\frac{{n_{{{\text{qn}}}} \left[ n \right]}}{{U_{{{\text{amp}}}} }} = n_{{{\text{qn}}}} \left[ n \right]$. (**b**) The PM signal is amplified to $u_{{{\text{amp}}}} \left( t \right)$ with $U_{{{\text{amp}}}} =$ 2 V and clipped to $u_{{{\text{amp}},{\text{clipped}}}} \left( t \right)$ with clipping threshold $K = 1 \;\mathrm{V}$. The clipped PM signal is synchronous sampled with $f_{{\text{s}}} = f_{{\text{c}}}$ and digitized to $u_{{{\text{digital}}}} \left[ n \right]$. The digitized demodulated results $\varphi_{{{\text{digital}}}} \left[ n \right]$ have a quantization error effect of $\frac{{n_{{{\text{qn}}}} \left[ n \right]}}{{U_{{{\text{amp}}}} }} = 0.5n_{{{\text{qn}}}} \left[ n \right]$ because of a larger $U_{{{\text{amp}}}} =$ 2 V. Thus, the error has been reduced by factor 2. (**c**) The PM signal is amplified to $u_{{{\text{amp}}}} \left( t \right)$ with $U_{{{\text{amp}}}} =$ 4 V and clipped to $u_{{{\text{amp}},{\text{clipped}}}} \left( t \right)$ with clipping threshold $K = 1 \;\mathrm{V}$. The clipped PM signal is synchronous sampled with $f_{{\text{s}}} = f_{{\text{c}}} /2$ and digitized to $u_{{{\text{digital}}}} \left[ n \right]$. The digitized demodulated results $\varphi_{{{\text{digital}}}} \left[ n \right]$ are obtained with a lower sampling frequency but a reduced by factor 4 quantization error effect of $\frac{{n_{{{\text{qn}}}} \left[ n \right]}}{{U_{{{\text{amp}}}} }} = 0.25n_{{{\text{qn}}}} \left[ n \right]$ because of a larger $U_{{{\text{amp}}}} =$ 4 V.

Our simulation results in Fig. 10 demonstrate that the resolution of the digitized demodulated results ${\varphi }_{\mathrm{digital}}[n]$ with clipping are significantly better than without clipping, if the quantization noise is the dominating noise component in the digital signal. Figure 10a illustrates a synchronous sampling and digitization without clipping by a 4-bit ADC. The quantization error ${n}_{\mathrm{qn}}$ can take a value between $\pm LSB/2$ with the least-significant-bit $LSB=0.125\; \mathrm{V}$ for a 4-bit ADC. The digitized demodulated results ${\varphi }_{\mathrm{digital}}[n]$ have the typical quantization error effect of $\frac{{n}_{\mathrm{qn}}\left[n\right]}{{U}_{\mathrm{amp}}}={n}_{\mathrm{qn}}\left[n\right]$, if the PM signal is amplified to ${U}_{\mathrm{amp}}=2 \;\mathrm{V}$ and clipped with clipping threshold $K = 1 \;\mathrm{V}$ as shown in Fig. 10b. Because the amplified modulated carrier signal ${u}_{\mathrm{amp}}\left(t\right)$ can be approximated at the sampling time points $t\left[n\right]$ as ${u}_{\mathrm{amp}}\left(t\left[n\right]\right)={U}_{\mathrm{amp}}\varphi \left(t\left[n\right]\right)$, the amplified information signal ${U}_{\mathrm{amp}}\varphi \left(t\right)$ is sampled synchronously with ${f}_{\mathrm{s}}={f}_{\mathrm{c}}$ and digitized directly. The quantization error effect of the digitized demodulation result ${\varphi }_{\mathrm{digital}}[n]$ is $\frac{{n}_{\mathrm{qn}}\left[n\right]}{{U}_{\mathrm{amp}}}=0.5{ n}_{\mathrm{qn}}\left[n\right]$, so the error is reduced by a factor 2. This results in a 6 dB improvement in signal-to-noise ratio of the digitized demodulation result ${\varphi }_{\mathrm{digital}}[n]$ relative to quantization noise in a demodulation bandwidth. The digitized demodulation result ${\varphi }_{\mathrm{digital}}[n]$ in Fig. 10c is obtained at a lower sampling frequency with ${f}_{\mathrm{s}}={f}_{\mathrm{c}}/2$, but the quantization error effect for ${\varphi }_{\mathrm{digital}}[n]$ is reduced by a factor of 4 due to $\frac{{n}_{\mathrm{qn}}\left[n\right]}{{U}_{\mathrm{amp}}}=0.25 {n}_{\mathrm{qn}}\left[n\right]$ with ${U}_{\mathrm{amp}}=4 \;\mathrm{V}$.

Although IQ demodulation, which is often used in communications and measurements, is also used to reduce the effects of random noise by a small degree of clipping, clipping may cause signal distortions by affecting the carrier and sidebands differently^33^. Therefore, when the signal is amplified to a strong saturation, the carrier-to-noise ratio of the digital signal is usually reduced because of the signal power loss and the generated higher-order harmonics in the digital signal. Detecting the timing of the zero crossings is also a phase demodulation technique, since the information is encoded in the zero-crossing point of the phase-modulated carrier signal. In order to obtain more accurate crossing times, amplification and clipping are also often applied before crossing detection. However, for a small phase $M$ modulated signal at a high frequency carrier ${f}_{\mathrm{c}}$, the time difference of zero crossings $\Delta t\le \frac{M}{2\pi {f}_{\mathrm{c}}}$ between modulated and unmodulated carrier is so small that a large error is introduced. In order to demodulate a bioelectrical impedance signal^43^ and prevent spectrum leakage, synchronous sampling is a common technique when converting analog to digital. In this paper, the sampling rate is exactly twice of the carrier frequency, and compared to the common synchronous sampling. We define the sampling point to fall precisely in the linear region of the clipped signal. Amplification of the signal increase the resolution at the critical sampling points and the saturated signal is not sampled. Thus, the digital signal does not contain harmonics and power losses for the presented method that contains clipping and synchronized sampling in the remaining linear signal segments. On the other hand, the sampling point at the carrier zero crossing converts the very small time difference into a quantizable amplitude by the slope at the zero crossing in respect to Eq. 7, so that the sideband information is recovered in the digital signal with full bit resolution while the carrier amplitude ${U}_{\mathrm{amp}}$ is defined by the AGC.

The advantage of our method is that the task of reducing digital noise is transferred to amplification and clipping design, rather than completely relying on the number of bits and oversampling rate of the ADC. Each clipping of half the amplitude of the PM signal ${u}_{\mathrm{amp}}\left(t\right)$ can achieve a 6 dB improvement for the SNR of the demodulated phase $\varphi \left[n\right]$ as shown in Fig. 10b. It is equivalent to the boost for the ADC of increasing by 1 bit number or 4 times oversampling. The method is suited for broad-bandwidth PM signals if the maximal phase stroke multiplied with the controlled amplitude of the AGC remains below the input range of the ADC. Thus, the method is able to improve the digital resolution of demodulation results limited by ADC dynamic range for measurement devices where information is stored in phase, e.g., LDV^37^, Doppler optical coherence tomography (DOCT)^44^ and surface plasmon resonance heterodyne phase interrogation sensor^45^. These measuring instruments offer relatively high sensitivity and resolution in measurement of parameters such as pressure, refractive index, displacement and strain. Moreover, the use of clipping and synchronous sampling can replace the high-resolution and high-frequency ADC in the field of PM signal receiver to effectively reduce the power consumption of the receiver.

In addition, we only require an ADC with a sampling frequency (${f}_{\mathrm{s}}=2{f}_{\mathrm{c}}$) lower than Nyquist sampling frequency (${f}_{\mathrm{s}}>2 {f}_{\mathrm{c}}+2{f}_{\mathrm{m},\mathrm{ max}}$) for a PM signal with the full modulation bandwidth defined by the Carson rule for the employed carrier frequency. Here, ${f}_{\mathrm{m},\mathrm{ max}}$ is the highest frequency component of the phase modulation $\varphi \left(t\right)$. On the other hand, for a band-limited PM signal, our method can also use a band-pass sampling frequency (${f}_{\mathrm{s}}=q{f}_{\mathrm{c}}>2{f}_{\mathrm{m},\mathrm{ max}}, q=\frac{1}{N}$), i.e., only one sample point at zero crossing of the carrier is acquired every *N* carrier cycles. Figure 10c shows the case of $q=\frac{1}{2}.$ Based on the use of a low sampling rate ADC, our method can also improve the sampling resolution by proper design of amplifying and clipping compared to the direct band-pass sampling. High dynamic range, broadband RF and microwave signals have a significant impact on military applications, cellular wireless sensor network architectures and RF sensor implementations^46^. Their carrier frequencies are located from GHz to more than a dozen GHz. Our method has the possibility to reduce the sampling rate limit to the band-pass sampling rate and to prevent the deterioration of the digital resolution by suitable amplification clipping. Therefore, the direct digitization becomes possible and provides a simplified concept for the structure of RF and microwave signal reception equipment.

Another advantage of our method is that demodulation is accomplished while sampling, and thus, a digital demodulation scheme can be skipped. The digitized signal as the demodulated results can be fed directly into the subsequent processing, e.g. filtering, signal processing algorithms, etc. We aim to achieve shot-noise limited demodulation of HI carrier signals and even sub-shot noise limited resolution could be possible by employing squeezed light.

The challenge of this method is a stable clock signal and synchronization as shown in Eq. (11). Nowadays, commercially available high performance ADCs must feature phase-jitter significantly below 1 ps RMS, the state-of -art ADCs such as AD9446 from Analog Devices with the aperture jitter ${t}_{\mathrm{aperture}}$ of 60 fs, ADC12DJ5200RF from Texas Instruments with aperture jitter ${t}_{\mathrm{aperture}}$ of 60 fs and the R&S®SMA100B RF and microwave signal generator provides a clean clock source with jitter of only ${t}_{\mathrm{clock}}=$ 20 fs RMS. This means that the voltage error is ${V}_{\mathrm{error}}={U}_{\mathrm{amp}}{2}\uppi {f}_{\mathrm{c}}{t}_{\mathrm{J}}=8 \;\mathrm{\mu V}$ caused by the phase jitter ${t}_{\mathrm{J}}=60 \;\mathrm{fs}$ with the carrier frequency ${f}_{\mathrm{c}}=40 \;\mathrm{MHz}$ and signal amplitude ${U}_{\mathrm{amp}}=500 \;\mathrm{mV}$. Such a low jitter noise is negligible in high performance ADCs compared to the thermal noise typically greater than $50 \;\mathrm{\mu V}$. The better the synchronization achieved between the sampling clock and the carrier signal, the less the influence generated by clock jitter. The application of the new method in heterodyne interferometry presents some special challenges. Optical turbulence or laser frequency drift can change the carrier frequency at low frequencies. The situation in our temperature-stabilized laboratory was stable enough to suppress such effects. In a more disturbing environment, the measurement beam after the Bragg cell could be directly superimposed onto a second balanced photo detector with the reference beam, without going through the measurement object and, thus, without carrying the relevant measurement information. This signal could serve as the synchronization signal in order to minimize jitter that was not present in our environment during the experiments. The same carrier excitation signal, optical path length and the experimental environment can ensure this signal is highly synchronized with the carrier in the modulated signal carrying the information. Another option is to use the method of recovering the carrier directly from the modulated signal to generate the sampling signal, for example, extracting the synchronization signal from the modulated signal with a narrow bandwidth filter.

In this paper, we give an application of our method to optical measurements using HI as an example. The experiment results of the digital demodulation of a PM signal with a small modulation index by using an 8-bit ADC demonstrate the achievable resolution for HI vibration amplitude measurements in Fig. 8. The smallest measurable phase deviation for this signal can be adjusted by our method to $23\;\mathrm{ fm}/\sqrt{\mathrm{Hz}}$ instead of $50\;\mathrm{ fm}/\sqrt{\mathrm{Hz}}$ limited by digital noise. Today, HI finds applications in various analytical fields for example, use of HI to characterize acoustic waves in water and in air by monitoring changes in the refractive index of water^47^, the use of refractive tomography to visualize acoustic field tomography in 3-dimensional gas and liquid volumes^48^ and the use of HI microscopy for high precision surface measurement^49^.

## Conclusion and outlook

This paper focuses on the problem of CNR degradation of small phase-modulated signals during digitization and proposes a simple sampling and demodulation method. We proved that it is possible to beat the quantization noise limitation of the PM signal with a small modulation index and a large CNR by employing clipping and proper synchronized sampling of the carrier signal.

We proposed a sampling method where the sampling rate is matched to an integer multiple of the carrier frequency and the zero points of the carrier are acquired in each period. This sampling method provides a more efficient use of the acquisition range of the ADC by the clipping in order to digitize the sidebands with a higher bit resolution. The gain of the carrier amplitude is no longer limited by the input range of the ADC because the carrier amplitude is fed into the demodulation scheme. In case of HI the carrier amplitude is well-defined by the AGC gain. The paper gives a new upper limit of the modulation index in dependence of digitization settings and jitter, where the PM signal can be obtained correctly. Consequently, signals with a smaller modulation index can be digitized with a greater amplification and signal power, if a good synchronization can be guaranteed.

In the paper, we demonstrated that the displacement resolution of a HI for a low bit numbers ADC can be improved from $50\;\mathrm{ fm}/\sqrt{\mathrm{Hz}}$ limited by digital noise to $23\;\mathrm{ fm}/\sqrt{\mathrm{Hz}}$ with clipping compared to conventional sampling technique without clipping. The technique has the potential to overcome the digital noise limitation and to achieve a shot-noise limited resolution for HI in the order of ${s}_{\mathrm{rl}}\approx 1\;\text{ fm/}\sqrt{\text{Hz}}$ or by employing squeezed-light techniques even in the attometer regime. However, our new method may have a much larger impact since it can be employed to demodulate all carriers with broad-bandwidth modulations where a limited modulation index with $M\ll 1$ is possible.

## Supplementary Information

Supplementary Information 1.

Supplementary Information 2.

Supplementary Information 3.

Supplementary Information 4.

Supplementary Information 5.

Supplementary Information 6.

Supplementary Information 7.

## Data Availability

All data generated or analysed during this study are included in this published article [and its supplementary information files].
