# Supplementary material for: Improved demodulated phase signal resolution for carrier signals with small modulation index by clipping and synchronous sampling for heterodyne interferometers
Source: Sci Rep. 2023 May 26;13:8570. doi: 10.1038/s41598-023-35000-2 (PMC10220044; doi:10.1038/s41598-023-35000-2)
Supplement: Supplementary file 2 — Supplementary Information 2. [file 41598_2023_35000_MOESM2_ESM.zip › Legend for Supplementary data D2.docx]

Supplementary data D2: Raw data for spectrum of the displacement measured by HI with RBW=1 Hz of the clipped signal when using the demodulation method with q=2.

This data is the time-dependent voltage signal collected by the acquisition card in volts. This signal is demodulated to obtain the displacement signal. Its spectrum, as shown in Figure 8 of the manuscript for the clipped signal.
