# Supplementary material for: Improved demodulated phase signal resolution for carrier signals with small modulation index by clipping and synchronous sampling for heterodyne interferometers
Source: Sci Rep. 2023 May 26;13:8570. doi: 10.1038/s41598-023-35000-2 (PMC10220044; doi:10.1038/s41598-023-35000-2)
Supplement: Supplementary file 3 — Supplementary Information 3. [file 41598_2023_35000_MOESM3_ESM.zip › Supplementary data D3/Legend for Supplementary data D3.docx]

Improving the resolution of demodulated phase signals for carrier signals with small modulation index by clipping and synchronous sampling for heterodyne interferometers

M. Yu*, M. Schewe, G. Bauer and C. Rembe

Supplementary data D3: Raw data for direct acquisition results of the 40 MHz signal generated by the signal generator. This value can be used when converting ADC values (in LSB=1/65535) into real-world voltage values. This signal demonstrates the disturbance components in the carrier signal.
